# Late Pennsylvanian carbonate platform facies and coral reef: new insights from southern China (Guizhou Province)

**DOI:** 10.1007/s10347-020-00613-w

**Published:** 2020-11-10

**Authors:** Marine Maillet, Wen-Tao Huang, Xiao Li, Zhen-Yuan Yang, Chang-Qing Guan, Yong-Li Zhang, En-Pu Gong, Katsumi Ueno, Elias Samankassou

**Affiliations:** 1 Department of Earth Sciences, University of Geneva, Rue des Maraîchers, 13, 1205 Geneva, Switzerland; 2 Department of Geology, Northeastern University, Wenhua Road 3-11, Heping district, Shenyang, 110819 Liaoning People’s Republic of China; 3 University of Fukuoka, 8-19-1 Nanakuma, Jonan-ku, Fukuoka, 814-0180 Japan

**Keywords:** Pennsylvanian, Kasimovian–Gzhelian, Coral reef, Carbonate platform

## Abstract

The Pennsylvanian is characterized by intense paleoenvironmental changes related to glacio-eustatic sea-level fluctuations and major tectonic events, which affected the evolution of biocommunities. Most known Pennsylvanian tropical reefs and mounds are predominantly composed of calcareous algae (e.g. phylloid algae, *Archaeolithophyllum*), calcareous sponges, fenestrate bryozoans, *Tubiphytes*, and microbialites. However, in Houchang (southern China), the Late Pennsylvanian carbonate platform records a large coral reef lacking any analogs in age (Gzhelian), size (80–100 m thick) and composition (high biodiversity). The large coral reef developed at the border of the Luodian intraplatform basin. The intraplatform basin is characterized by the deposition of green algal grainstone, coated grain grainstone and bioclastic packstone, grainstone, floatstone and rudstone in shallow-waters. In the deep-water shelf, lithofacies are composed of burrowed bioclastic wackestone, microbioclastic peloidal packstone, grainstone, and fine-grained burrowed wackestone and packstone. In this context, the coral reef developed on a deep-shelf margin, in a moderate to low energy depositional environment, below the FWWB. The scarcity of Pennsylvanian coral reefs suggests global unfavorable conditions, which can be attributed to a complex pattern of several environmental factors, including seawater chemistry (aragonite seas), paleoclimatic cooling related to continental glaciation, and the biological competition with the more opportunistic and adaptive phylloid algal community that occupied similar platform margin paleoenvironments. The existence of the large Bianping coral reef in southern China, as well as a few additional examples of Pennsylvanian coralliferous bioconstructions, provides evidence that coral communities were able to endure the Late Paleozoic fluctuating paleoenvironmental conditions in specific settings. One of such settings appears to have been the deep shelf margin, where low light levels decreased competition with the phylloid algal community.

## Introduction

The Carboniferous is a critical interval in Earth History, characterized by major cyclic paleoenvironmental changes such as climate, seawater chemistry and high frequency glacio-eustatic sea-level fluctuations, which directly influenced the evolution and development of biological communities (Brand 1989; Heckel et al. 2007; Heckel 2008; Davydov et al. 2012; Wang et al. 2013). The Mississippian is considered as a period of metazoan reef-building collapse subsequent to the Late Devonian extinction events (Sandberg et al. 1988; Thompson and Newton 1988; Buggisch 1991; Webb 2002; Kaiser et al. 2015; Barash 2017). During the Pennsylvanian and Permian, new types of organic carbonate buildup communities evolved that were composed of various combinations of calcareous algae, bryozoans, calcareous sponges and microbialites (Copper 1986, 2002; West 1988; Wahlman 2002; Webb 2002). Sparse Carboniferous shallow-water buildups containing corals have been described, but corals generally played only minor roles in their construction (Wilson 1963; Wahlman 2002; Ogar 2012).

Sparse large Pennsylvanian coral-rich reefs have been described, such as the Bashkirian–Moscovian reefs of the Akiyoshi Group in Japan, which contain abundant tabulate and rugose corals (Ota 1968; Nagai 1985; Fagerstrom 1987; Sugiyama and Nagai 1990, 1994; Nakazawa 1997; Wahlman 2002), and the Kasimovian–Gzhelian coral reef (80–100 m thick and 700 m wide) reported in Guizhou, southern China (Zhang et al. 2010; Gong et al. 2012). The well-developed coral reef reported in Bianping (Ziyun County, Guizhou) is considered to be the largest Pennsylvanian organic carbonate buildup with a coralliferous reefal framework in the world (Zhang et al. 2010). To understand the overall context leading to the development of this exceptional reef growth (platform architecture and environmental conditions), two sections located in Zhongxinzhai and Brickyard villages, respectively (Houchang, Ziyun, Guizhou), have been investigated, and selected samples have been analyzed for petrographic characteristics, biostratigraphic age (fusulines), and chemostratigraphy (Sr isotopes). The existence of the large Pennsylvanian coral reef, which persisted only in a specific setting as shown in the present study, holds the potential to improve our current understanding of the collapse and recovery of coral reefs.

## Geological settings

### Southern China paleogeography

**Fig. 1:**
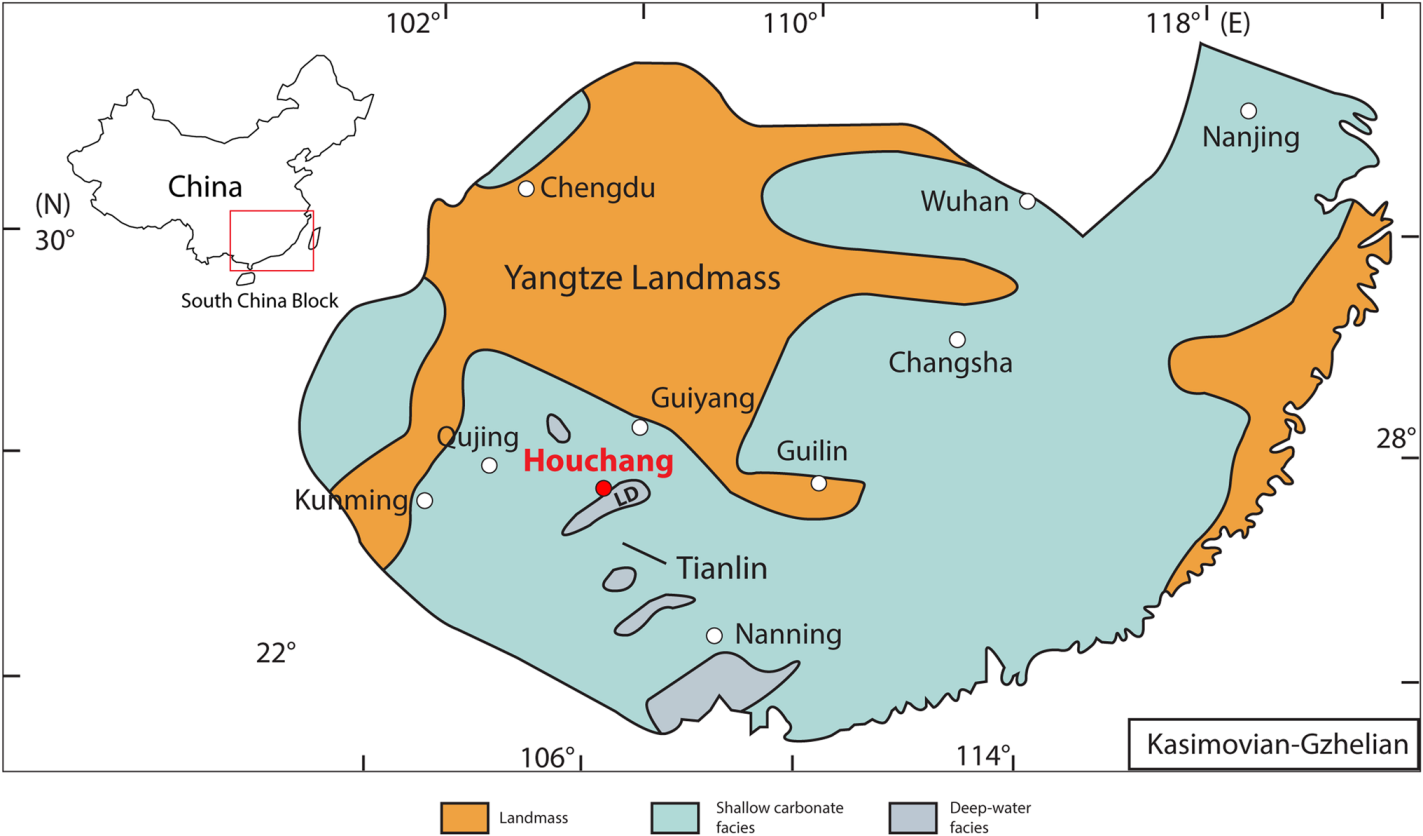
Late Pennsylvanian paleogeographic map of southern China (modified from Feng et al. 1998; Yao and Wang 2016). Ziyun (Guizhou) is the study area. *LD* Luodian Basin

During the Pennsylvanian and Early Permian (Asselian–Sakmarian), in the Guangxi Autonomous Region and Guizhou Province (southern China), sedimentation occurred in the Dian–Qian–Gui Basin at the passive continental margin of the Yangtze Craton. During the Carboniferous, the Dian–Qian–Gui Basin records several elongate depressions (SW–NE axis), separated by more broad structural highs, resulting from pre-Carboniferous syndepositional faulting (Tsien et al. 1988; Shen 2002). During the Late Pennsylvanian–Early Permian (Asselian), the area consisted of a tropical epicontinental sea, dominated by carbonate platform facies associated with intraplatform depression facies (Shen and Qing 2010; Wang et al. 2013; Fig. 1). During the latest Sakmarian, a major sea-level fall occurred, which produced a series of coal-bearing strata and an extensive sedimentary hiatus throughout South China (Wang et al. 2013).

### Study area

**Fig. 2:**
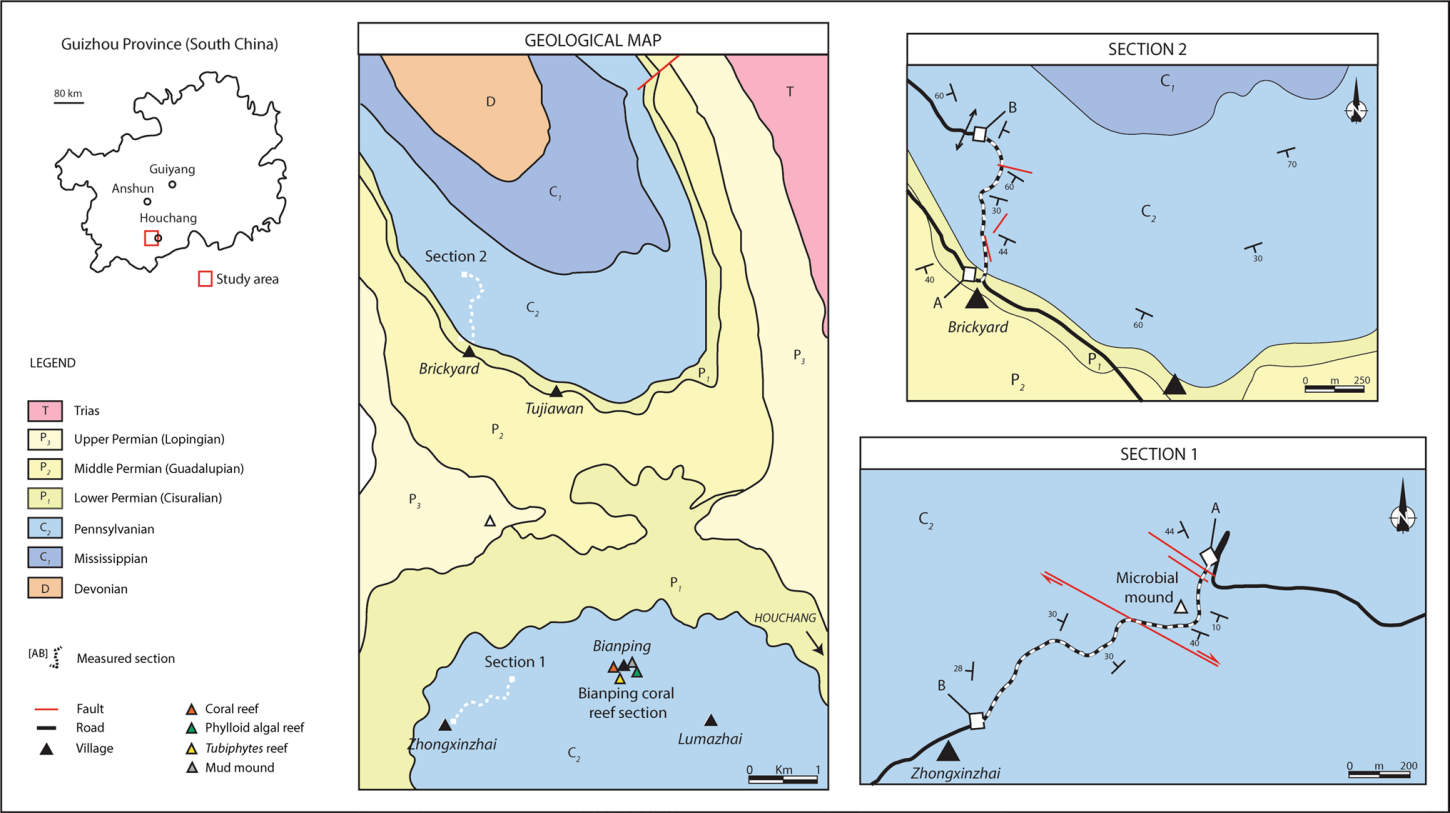
Geological map of the Houchang area (Ziyun, Guizhou), showing locations of measured sections 1 and 2. On the right side are closer maps showing the paths of the two sections, with the bases of the measured sections are in A, and the tops of the sections in B

The study area around the town of Houchang (Ziyun County, Guizhou Province) exhibits folded Devonian to Triassic carbonate rock units (Fig. 2). During the Carboniferous, the Houchang area was located at the margin of the Luodian intraplatform basin (Fig. 1), where numerous bioconstructions have been reported (Yao and Wang 2016).

Late Pennsylvanian bioconstructions include microbial mounds (Huang et al. 2019), sponge reefs (Tan 1991; Yao and Wang 2016), phylloid algal reefs (Gong et al. 2007a, b), *Tubiphytes* reefs (Chang et al. 2008; Guan et al. 2010; Yao and Wang 2016), a small mud mound (Chang et al. 2008; Gong et al. 2012) and a large coral reef (Zhang et al. 2010; Gong et al. 2012). All of these bioconstructions were roughly interpreted as growing at the platform margin (Table 1), but the sedimentary context was not constrained.

**Table 1:** A synthesis of bioconstructions, reported around the Houchang town

	Microbial mounds	Sponge reef	*Tubiphytes* reef	Phylloid algal reef	Carbonate mud mound	Coral reef
Age	*Late Moscovian*	*Late Kasimovian–Gzhelian*	*Gzhelian*	*Gzhelian*	*Gzhelian*	*Gzhelian*
Number	2	14	> 3	> 10	1	1
Size	Wide: 30–200 m Height: 15–19 m	Wide: 100–200 m Height: 8–12 m	Wide: 0.7–3 m Height: 0.3–0.5 m	Wide: 30–50 m Height: 2–8 m	Wide: 2.5 m Height: 1 m	Wide: 700 m Height: 80–100 m
Main reef-builder	Unknown microbes	*Solenopora*	*Tubiphytes*	Phylloid algae	Calcimicrobes (blue-green algae)	*Fomitchevella* associated to phylloid algae, calcimicrobes, *Ivanovia* cf *manchurica* and *Antheria*
Associated organism	Tube-shaped algae, crinoid, bryozoans, brachiopods, corals, chaetetids, phylloid algae, *Ivanovia* and calcimicrobes	Crinoids, brachiopods, fusulinids, bryozoans, solitary rugose corals, green algae	Fusulinids	Brachiopods	Crinoids	Brachiopods, fusulinids, foraminifera, crinoids, algae, bryozoans, *Tubiphytes*
Sedimentary facies	Reef-core: microbial boundstone Underlying: bioclastic wackestone and packstone	Reef-core: *Solenopora* framestone and bindstone	Overlying: bioclastic grainstone Reef-core: *Tubiphytes* bindstone Underlying: Bioclastic grainstone	Overlying: bioclastic packstone and grainstone Reef-core: Phylloid algae bafflestone and framestone Underlying: Bioclastic packstone and grainstone	Overlying: bioclastic grainstone Reef-core: blue-green algae bindstone Underlying: bioclastic packstone	Overlying: bioclastic packstone and grainstone Reef-core: coral framestone, bindstone, and phylloid algal bafflestone Underlying: Bioclastic wackestone
Paleo-environment	Shallow platform margin	Shallow platform margin	Shallow platform margin	Shallow platform margin	Deep platform margin	Platform margin
Location	Zhongxinzhai and Lumazhai villages, Houchang town, Ziyun County, Guizhou Province	Wengdao village, Houchang town, Ziyun County, Guizhou Province	Bianping village, Houchang town, Ziyun County, Guizhou Province	Wengdao and Bianping villages, Houchang town, Ziyun County, Guizhou Province	Bianping village, Houchang town, Ziyun County, Guizhou Province	Bianping village, Houchang town, Ziyun County, Guizhou Province

Microbial mounds: Huang et al. (2019); Sponge reefs: Tan (1991), Yao and Wang (2016); Phylloid algal reefs: Gong et al. (2007a, b); *Tubiphytes* reefs: Chang et al. (2008), Guan et al. (2010), Yao and Wang (2016); Mud mound: Chang et al. (2008), Gong et al. (2012); Coral reef: Zhang et al. (2010), Gong et al. (2012)

The Late Moscovian microbial mounds (Zhongxinzhai and Lumazhai villages, Houchang), measuring 30–200 m wide and 15–19 m thick, are composed of microbial boundstone characterized by stromatolitic structures, irregular oncoid-like forms and wrinkle structures (unknown microbes). Microbial boundstone co-existed with tube-shaped algae, crinoids, bryozoans, brachiopods, corals (*Ivanovia*, not to be confused with the synonymous phylloid alga common in Pennsylvanian deposits), chaetetids, phylloid algae, and numerous calcimicrobes including *Girvanella*, *Ortonella*, *Wetheredella*-like, *Palaeomicrocodium*-like, and some problematic calcimicrobes (Huang et al. 2019). The microbial mounds might have formed in the euphotic zone, with shallow-water, normal salinity, and under relatively high-energy conditions (Huang et al. 2019).

The Late Kasimovian–Gzhelian calcisponge reefs (Wengdao village, Houchang) are 100–200 m wide and 8–12 m thick. Reef-building organisms are dominated by chaetetid sponges (*Solenopora*), associated with green algae, solitary rugose corals, bryozoans, brachiopods, crinoids and foraminifera. The calcareous sponge reefs are currently interpreted as growing at a very shallow platform margin (Tan 1991; Yao and Wang 2016).

The Gzhelian phylloid algal reefs (Wengdao and Bianping villages, Houchang) are 30–50 m wide and 2–8 m thick. They are built mainly by phylloid algae, characterized by single cup-shaped, cabbage-shaped and clustering cup-shaped morphologies, associated to brachiopods, foraminifera, corals, crinoids, bryozoans, gastropods and *Tubiphytes* (Fan and Rigby 1994; Gong et al. 2007a, b; Guan et al. 2010; Gong et al. 2012; Yao and Wang 2016). These phylloid algal reefs developed below the FWWB, in a moderate energy environment, within the photic zone (Gong et al. 2007a, b).

The Gzhelian *Tubiphytes* patch reefs (Bianping village, Houchang) are 0.7–3 m wide and 0.3–0.5 m thick and are dominated by *Tubiphytes* (Guan et al. 2010), which might represent calcimicrobes during this time (Flügel 2010; Yao and Wang 2016). These reefs were interpreted to have grown at a shallow platform margin (Yao and Wang 2016).

The Gzhelian carbonate mud mound (Bianping village, Houchang) is built mainly by calcimicrobes (Yao and Wang 2016) associated with crinoids. It measures 2.5 m width and 1 m thickness. The mud mound was interpreted as growing at a deeper platform margin (Yao and Wang 2016).

**Fig. 3:**
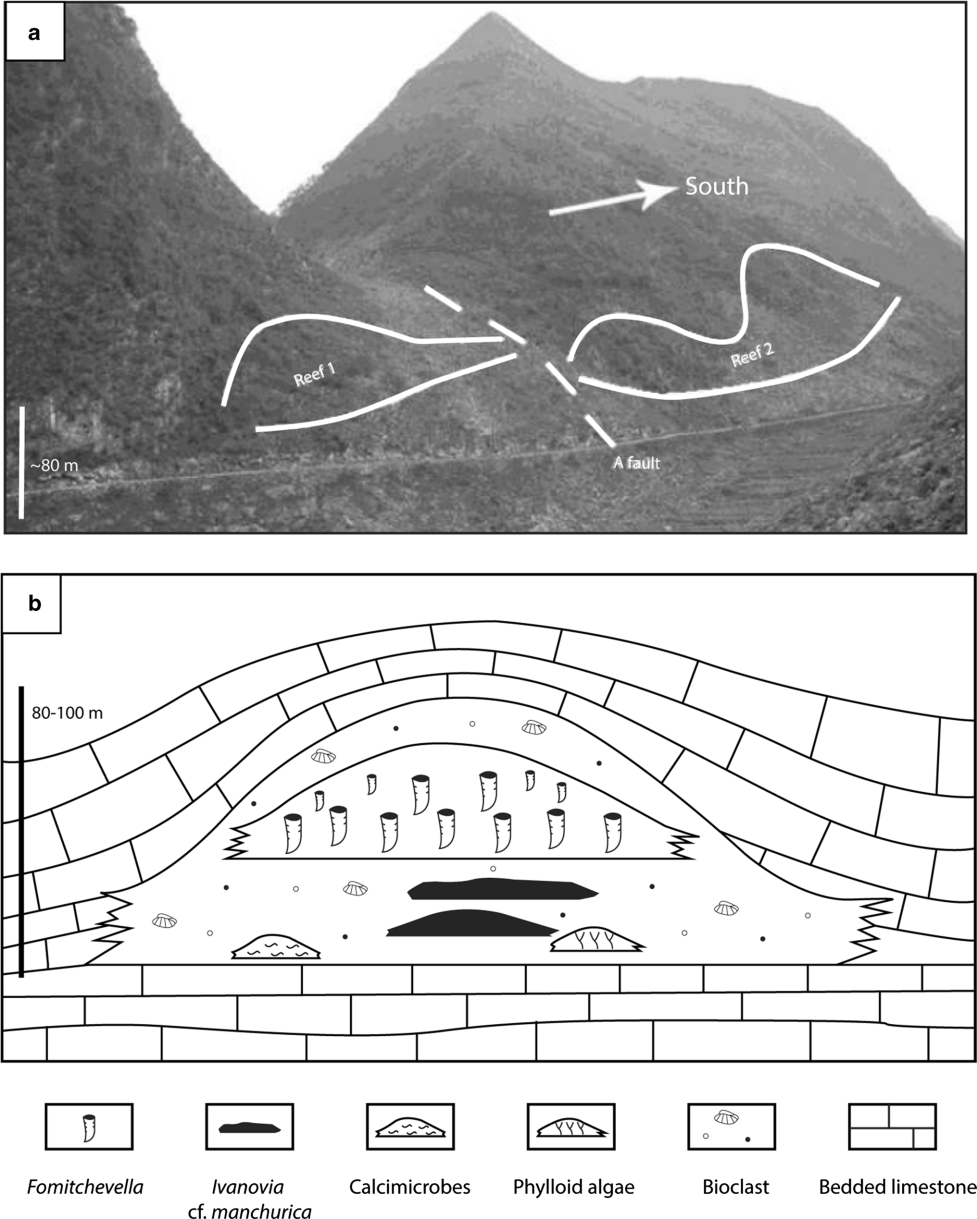
**a** Outcrop of the Bianping coral reef. The two exposures (Reef 1 and Reef 2) likely represent the same reef complex, separated by an apparent fault. **b** Sketch diagram of the Bianping coral reef showing the composition and distribution of reef-building biota and associated bioclastic facies. Modified from Gong et al. (2012)

The Gzhelian coral reef (Bianping village, Houchang) is 80–100 thick and about 700 m wide, and was constructed by large branching rugose colonial corals (*Fomitchevella* sp.)*.* Other reef-builders, such as phylloid algae, the corals *Ivanovia* cf. *manchurica* and *Antheria* sp., and microbialites constructed an initial reef facies and substrate for the branching corals to colonize (Fig. 3). Associated organisms include brachiopods, fusulines (*Triticites* and few *Quasifusulina*; Gong et al. 2004; Guan et al. 2004; Zhang et al. 2010; Gong et al. 2012), other undetermined smaller foraminifera, crinoids, algae, bryozoans and *Tubiphytes* (Zhang et al. 2010; Gong et al. 2012). Currently, the depositional environment of the Bianping coral reef is not well constrained.

### Global paleoclimate and glacio-eustatic fluctuations

The Carboniferous is commonly regarded as a time of global greenhouse–icehouse climate transition. Recent studies suggest multiple pulses of glaciation and deglaciation (Fielding et al. 2008a), with resulting high-frequency sea-level fluctuations (Ross and Ross 1987, 1988; Haq and Schutter 2008).

#### Late Paleozoic Ice Age

The Late Paleozoic Ice Age (LPIA) began with short-lived Mississippian (Tournaisian and Late Viséan) localized glacial events (e.g. South America, Isbell et al. 2003; Caputo et al. 2008; Fielding et al. 2008a; Henry et al. 2008). The onset of widespread glaciation occurred at the Viséan–Serpukhovian boundary in Gondwanaland (present-day South America and eastern Australia, Fielding et al. 2008a). During the earliest Bashkirian, Icehouse conditions expanded further, becoming widespread across South America (Holz et al. 2008; Rocha-Campos et al. 2008; Henry et al. 2008) and Australia (Fielding et al. 2008b; Mory et al. 2008). Further expansion of continental glacial ice centers in Southern Africa (Stollhofen et al. 2008; Isbell et al. 2008), Oman and Saudi Arabia (Martin et al. 2008) occurred at the Bashkirian–Moscovian boundary. During the early late Pennsylvanian, isotopic and paleobotanical records suggest a period of relative climatic warming (Frank et al. 2008; Pfefferkorn et al. 2008). Then, the latest Pennsylvanian–earliest Permian interval corresponds to a widely expanded glacial phase, with the growth of large ice sheets across Gondwana and accumulation of ice in the northern hemisphere (Rygel et al. 2008). Ice sheets are inferred to have been at their maximum extent around the Pennsylvanian–Permian boundary and persisted until the Late Sakmarian (Rygel et al. 2008), focused on Antarctica, Australia, southern Africa and South America (Fielding et al. 2008a).

#### Glacio-eustatic fluctuations

Throughout the Pennsylvanian, glacio-eustatic fluctuations are of high amplitude, varying from 10 to ~ 120 m (Ross and Ross 1987, 1988; Maynard and Leeder 1991; Haq and Schutter 2008; Rygel et al. 2008; Davydov et al. 2012), with a periodicity within the Milankovitch ranges (Harrison et al. 1979; Ross and Ross 1987; Heckel et al., 2007). In detail, from the latest Viséan–earliest Pennsylvanian, strata exhibit evidence of glacio-eustasy of 40–100 m (Rygel et al. 2008). Conversely, during the Mid-Pennsylvanian, glacio-eustatic fluctuations were less than 40 m, prior to reaching magnitudes of 100–120 m during the Late Pennsylvanian–earliest Permian (Rygel et al. 2008).

## Methods

### Petrography

**Fig. 4:**
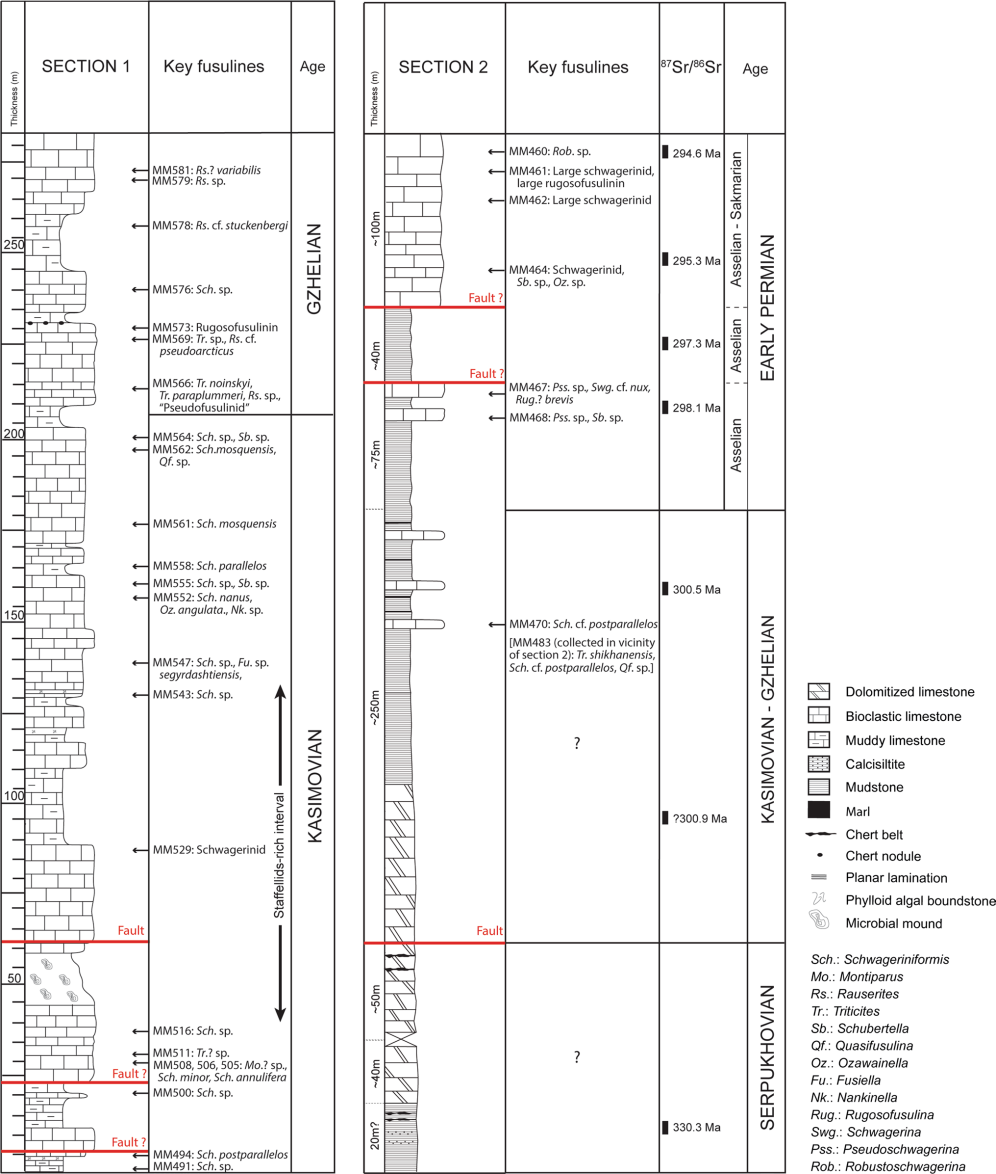
Measured sections 1 (Zhongxinzhai) and 2 (Brickyard) showing the lithofacies successions, the stratigraphic distribution of age-diagnostic key fusuline foraminifera, strontium absolute age dates (section 2 only), and chronostratigraphy. Section 1 is through relatively shallow-water ramp facies, and section 2 is through a relatively deeper water slope to basin succession

The dataset consists of three stratigraphic sections. Two sections were measured in the Zhongxinzhai (section 1) and Brickyard villages section 2; Fig. 4), respectively, which are five km apart. The third section was measured along the large Bianping coral reef (Gong et al. 2004, 2012; Zhang et al. 2010).

Thin sections from 123 samples were analyzed using a petrographic microscope. Lithofacies were classified following Dunham (1962) and Embry and Klovan (1971) and grouped according to grain assemblages and texture to interpret the depositional environments.

### ^87^Sr/^86^Sr dating

Twenty-eight carbonate samples were selected for Sr isotope analyses. Powders of bulk samples were collected, avoiding veins during drilling. The ratios were measured using a Thermo Neptune PLUS Multi-Collector inductively coupled plasma mass spectrometer, in static mode (University of Geneva). The method follows that described in Samankassou et al. (2018). Due to a systematic difference between measured and nominal standard ratios of the SRM987 of ^87^Sr/^86^Sr (0.710248), the internally corrected ^87^Sr/^86^Sr values were additionally corrected by a value of − 0.025‰ per amu for external fractionation (McArthur et al. 2001).

### Fusuline biochronology

A total of 46 fusulinid samples from section 1 and seven samples from section 2 were investigated to establish fusulinid-based chronostratigraphic framework (Fig. 4). Fusulines are the most important biostratigraphic index fossils used in this analysis, and were recently calibrated with high-precision U–Pb zircon absolute age-dates in the Carboniferous successions of the Donets Basin and Urals (Davydov et al. 2010; Schmitz and Davydov 2012).

## Age

### Section 1 (Zhongxinzhai)

**Fig. 5:**
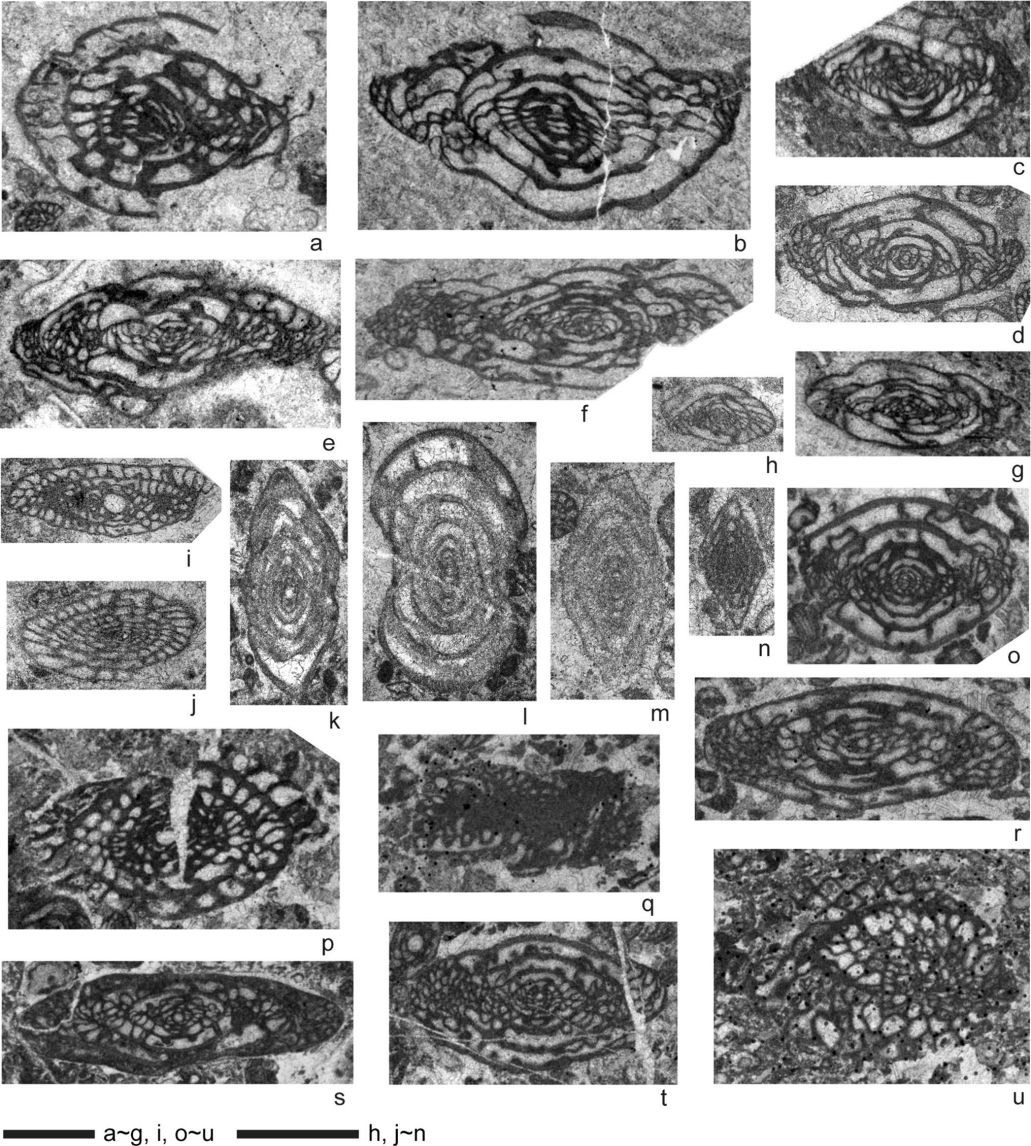
Representative age-diagnostic fusulines from section 1 (Zhongxinzhai). **a** *Montiparus*? sp., nearly tangential section, sample MM508. **b** *Schwageriniformis postparallelos* (Scherbovich), tangential section, sample MM494B. **c** *Schwageriniformis mosquensis* (Rozovskaya), axial section, sample MM561. **d** *Schwageriniformis nanus* (Rozovskaya), axial section, sample MM552. **e** *Schwageriniformis parallelos* (Scherbovich), axial section, sample MM505. **f** *Schwageriniformis annulifera* (Rauzer-Chernousova), axial section, sample MM505. **g** *Schwageriniformis minor* (Rozovskaya), axial section, sample MM506. **h** *Schubertella* sp., axial section, sample MM555. **i** *Quasifusulina* sp., axial section (immature specimen), sample MM562. **j** *Fusiella* cf. *segyrdashtiensis* Davydov in Leven and Davydov (2001), oblique section, sample MM547. **k**–**m** Indet. staffellids (k: *Reitlingerina*? sp., l: *Parastaffelloides*? sp., m: *Nankinella* sp.), all axial sections, k, l: sample MM517, m: sample MM552. **n** *Ozawainella angulata* (Colani), axial section, sample MM552. **o** *Triticites paraplummeri* Bensh, axial section, sample MM566. **p** *Rauserites* cf. *pseudoarcticus* (Rauzer-Chernousova), oblique section, sample MM569. **q** Indet. “pseudofusulin” (potentially *Ruzhenzevites* or *Andersonnites*), tangential section (fragment), sample MM566. **r** *Triticites noinskyi* Rozovskaya, axial section, sample MM566. **s** *Rauserites*? *variabilis* Rozovskaya, axial section, sample MM581. **t** *Rauserites* cf. *stuckenbergi* (Rauzer-Chernousova), axial section, sample MM578. **u** Indet. rugosofusulin (potentially *Dutkevitchia*), diagonal section, sample MM573. Scale bars equal to 1 mm

Fusulines occur throughout the Zhongxinzhai section (Figs. 4, 5). The lower, about 200 m-thick succession in this section is characterized by the occurrences of species of *Schwageriniformis,* including *S. minor*, *S. annuifera*, *S. nanus*, *S. parallelos*, *P. postparallelos*, and *S. mosquensis* (Fig. 5b–g). Similar *Schwageriniformis*-dominant assemblages have been widely reported from the middle–late Kasimovian of the Tethyan region (e.g. Leven and Davydov 2001; Leven 2009; Orlov-Labkovsky and Bensh 2015). From 40 to 133 m above the base of the section, schwagerinid fusulines are rare and staffellid fusulines are common, indicating more inner shelf, restricted marine paleoenvironments (Fig. 5k, l). From 220 m upsection, faunal associations change significantly. *Schwageriniformis* becomes rare and instead *Rauserites* becomes more common, including *R.* cf. *stuckenbergi*, *R*. cf. *pseudoarcticus*, and *R*.? *variabilis* (Fig. 5p, s, t). *Triticites paraplummeri* and *T. noynskyi* are also associated with them (Fig. 5o, r). Though represented by poor specimens in sections, there also occur some larger schwagerinids that are somewhat similar to *Ruzhenzevites* or *Andersonnites* (Fig. 5q) and *Dutkevitchia* (Fig. 5u). These forms are indicative of a Gzhelian age in a broad sense (e.g., Leven 2009; Orlov-Labkovsky and Bensh 2015). Therefore, fusulines indicate that section 1 ranges in age from the middle Kasimovian to Gzhelian (Fig. 4).

### Section 2 (Brickyard)

**Fig. 6:**
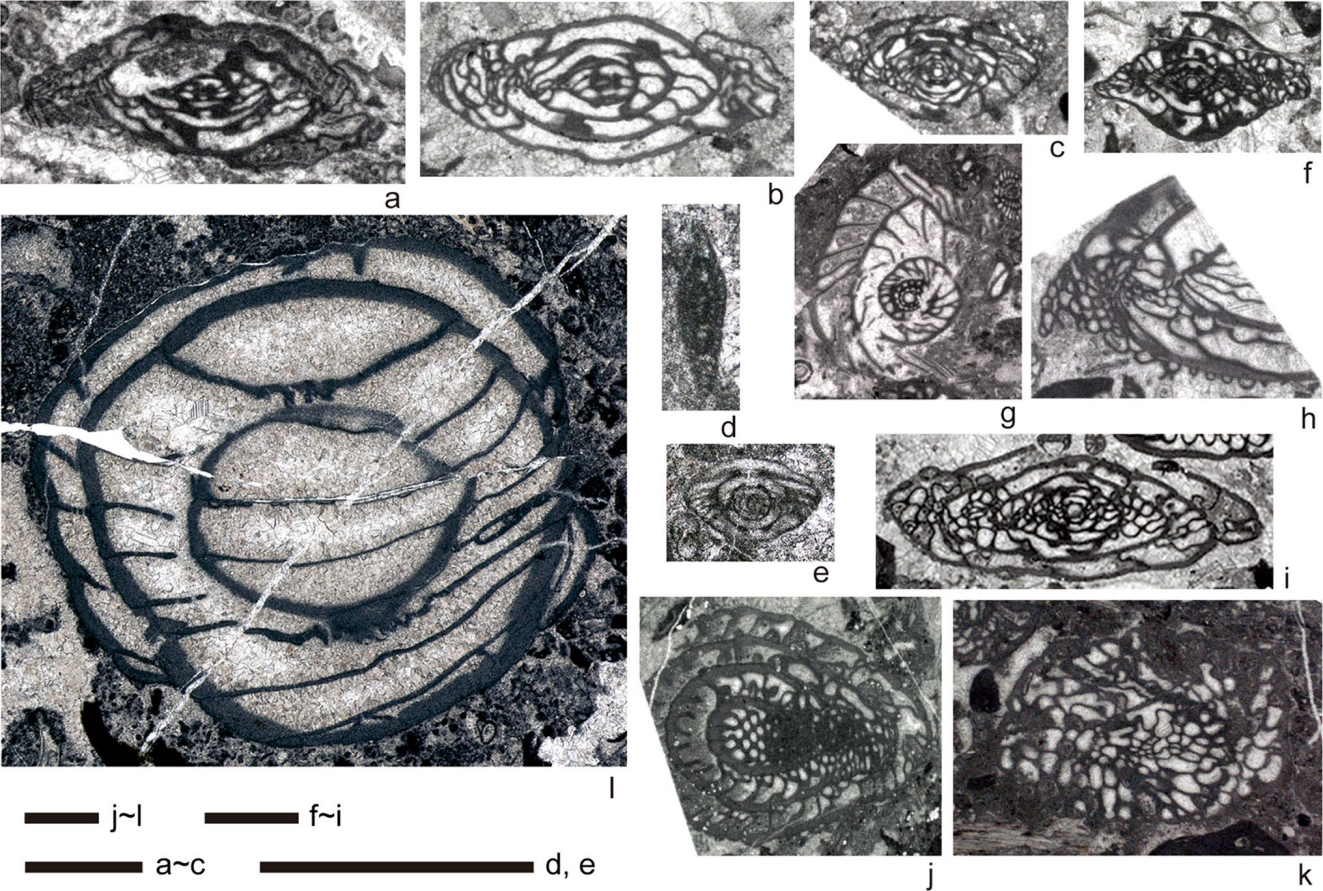
Representative age-diagnostic fusulines from section (Brickyard). **a**, **b** *Schwageriniformis* cf. *postparallelos* (Scherbovich), tangential sections, a: sample MM470, b: sample MM483. **c** *Triticites shikhanensis* Rozovskaya, axial section, sample MM483. **d** *Ozawainella* sp., tangential section, sample MM464, **e** *Schubertella* sp., axial section, sample MM464. **f** *Schwagerina* cf. *nux* (Schellwien), axial section (immature specimen?), sample MM467. **g**, **h** *Pseudoschwagerina* sp., g: sagittal section, sample MM468, h: tangential section (fragment), sample MM467. **i** *Rugosofusulina brevis* Leven and Scherbovich, axial section, sample MM467. **j** Indet. schwagerinid (“*Pseudofusulina*”), diagnonal section, sample MM462. **k** Indet. rugosofusulinin (probably advanced *Dutkevitchia*), diagonal section, sample MM461. **l** *Robustoschwagerina* sp., tangential section, sample MM460. Scale bars equal to 1 mm

Samples collected at Brickyard are poor in fossil content compared to the Zhongxinzhai section, with fusulines occurring at only a few horizons (Figs. 4, 6). Especially in the lower part of the section, carbonates are entirely dolomitized, which hampered biostratigraphic age-dating. Therefore, dating for this section relied on both Sr isotope chemostratigraphy and fusuline biochronology. The ^87^Sr/^86^Sr ratios range from 0.70798 to 0.70791. ^87^Sr/^86^Sr values from some key samples are summarized in Table 2. These results indicate ages ranging from the Serpukhovian (330 Ma) to Asselian (earliest Permian) (296 Ma; Howarth and McArthur 1997; McArthur et al. 2001, 2012). In the lower part of section 2, where fossils occurrences are poor, Sr isotope analysis was more important than biostratigraphy, but age dates based on isotopes and biostratigraphy consistently agreed in the middle and upper parts of the section (Fig. 4).

**Table 2:** Sr isotope values, from section 2 (Gradstein et al. 2012; McArthur et al. 2012; Wang et al. 2018)

Section 2 (Brickyard)				
	Sample	^87^Sr/^86^Sr	Age	
1	MM460	0.70791	294.6 Ma	Asselian
2	MM463	0.70795	295.3 Ma	Asselian
3	MM465	0.70803	297.3 Ma	Asselian
4	MM468	0.70806	298.1 Ma	Asselian
5	MM469	0.70814	300.5 Ma	Gzhelian
6	MM472	0.70815	300.9 Ma	Gzhelian
7	MM477B	0.70798	330.3 Ma	Serpukhovian

In the lower part of section 2, sample MM477B gives an age of ca. 330 Ma based on Sr isotope geochronology. The data show that this stratigraphic interval probably corresponds to the Serpukhovian. Two samples (MM470 and MM483; the latter was collected in the vicinity of section 2) from the middle of the section contain *Triticites shikhanensis*, *Schwageriniformis* cf. *postparallelos*, and *Quasifusulina* sp. (Fig. 6a–c). This assemblage is similar to that seen in the Kasimovian part of section 1. Moreover, a Gzhelian age (300.5 Ma) is obtained from sample MM469 by the Sr isotope dating. Thus, the middle part of section 2 is correlated to the Kasimovian and possibly to the Gzhelian. Higher up the section, two samples (MM467 and MM468) collected from thin limestone intervals contain *Pseudoschwagerina* sp., *Rugosofusulina brevis*, *Schwagerina* cf. *nux*, and others (Fig. 6f–i). An Asselian age is demonstrated for the limestones by these fusulines (Leven and Scherbovich 1978; Leven 2009). In the uppermost ~ 100 m-thick part of section 2, represented by bioclastic limestone, four samples were examined for age determination by fusulines. Sample MM464 yields schwagerinids and *Ozawainella* sp. (Fig. 6d). Although the youngest stratigraphic occurrence of the genus *Ozawainella* has not been much discussed among fusuline workers until now, available data suggest that its last occurrence is in the Asselian (Leven and Scherbovich 1978; Leven et al. 1992). This implies that the level of sample MM464 is pre-Sakmarian. Above this level, a large schwagerinid having a peculiar style of septal fluting occurred in sample MM461 (Fig. 6k). Although the thin-section of the specimen is very poor, it is probably referable to an advanced large *Dutkevitchia* species, such as *D. complicata*, *D. splendid*a, or *D. devexa*, all of which have large shells with characteristic irregular-shaped bubble-like septal flutings in the axial ends of their tests. Just above, sample MM460 contains a large spherical fusulinid with loose coiling, a thick spirotheca, and almost unfluted septa that is probably referable to *Robustoschwagerina* (Fig. 6l**)**. Based on these lines of evidence, a Sakmarian age is concluded for the uppermost part of section 2 (e.g. Leven and Scherbovich 1978; Kobayashi and Altiner 2008; Leven 2009). The overall age assessment based on fusulines in section 2 is in good agreement with the Sr chemostratigraphic data. The latter also suggests an earliest Permian age (Asselian) for the upper part of the section (Table 2). Therefore, section 2 can be correlated from the Serpukhovian to the lowermost Permian although a lower–middle Pennsylvanian interval is not clearly recognized (Fig. 4).

## Lithofacies types

### Green algal grainstone

**Fig. 7:**
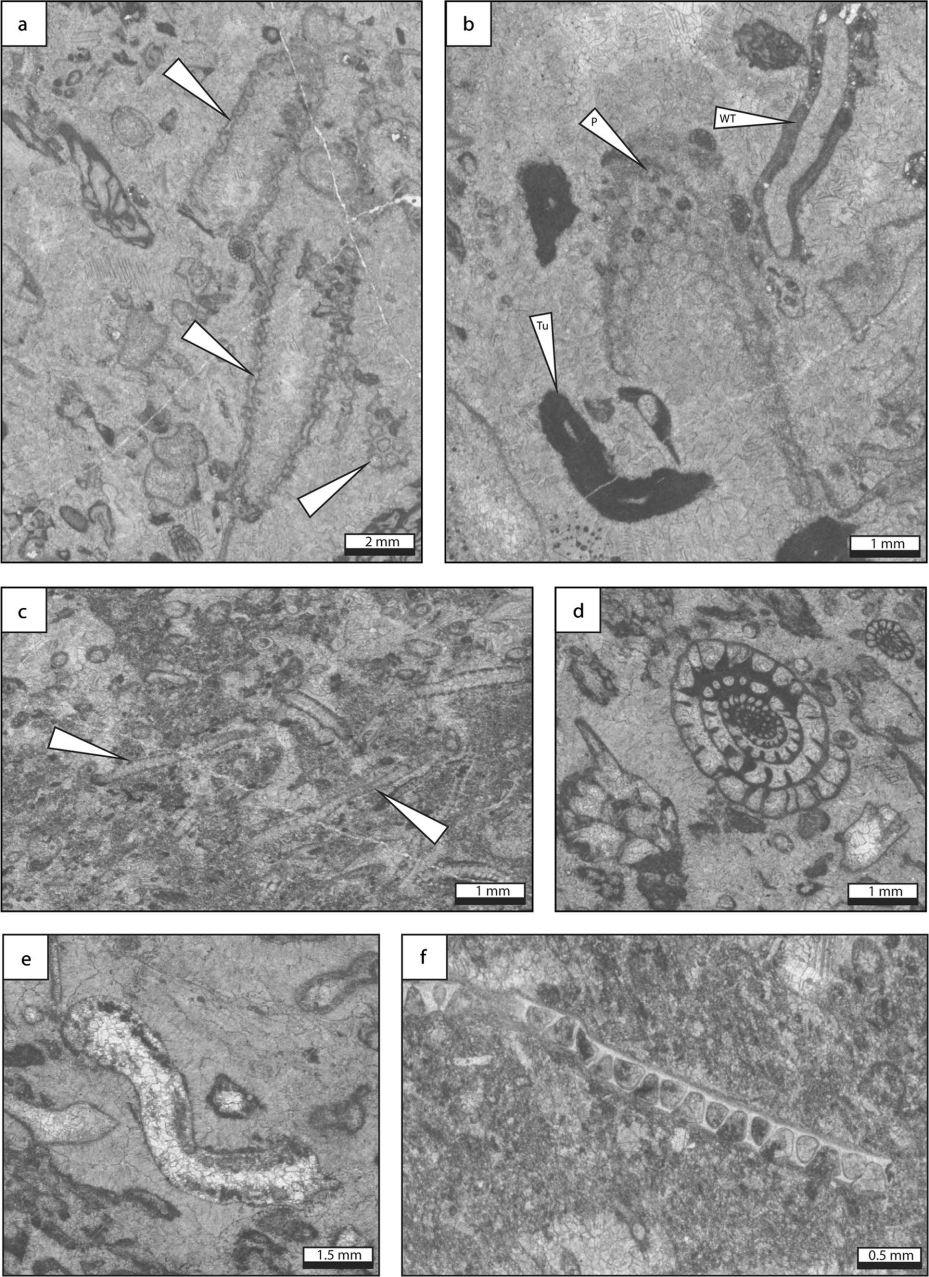
Green algal grainstone lithofacies type, which is the most updip and shallow-water facies type. **a** Dasycladacean green alga *Pseudogyroporella*-rich grainstone. **b** Green algal fragment with associated *Tubiphytes* and a worm tube. The cement consists of radiaxial fibrous and late blocky cement. **c** Poorly-washed grainstone with common *Beresella* algal fragments. **d** Oblique specimen of the fusuline *Schwageriniformis* sp. in bioclastic grainstone. **e** Phylloid algal fragment (center). **f** Fenestrate bryozoan fragment in fine-grained skeletal–peloidal packstone matrix. P: *Pseudogyroporella* sp., Tu: *Tubiphytes* sp., *WT* worm tube

*Description* Green algal grainstone is characterized by abundant *Pseudogyroporella* sp. and *Beresella* sp. that are commonly associated with foraminifera (*Triticites*, staffellids, schwagerinids, *Rauserites*) and echinoderms (Fig. 7). Gastropods, *Tubiphytes* sp., fenestrate bryozoans, brachiopods, phylloid algae and worm tubes (*Thartharella* sp.) represent minor components. Peloids, coated grains, mud clasts as well as grain aggregates are common. Grains range in size from 0.2 to 7 mm. Intergranular porosity is infilled by radiaxial-fibrous, drusy and blocky calcite cements. Locally, the green algal grainstone lithofacies is poorly washed, containing both micritic matrix and calcite cement.

*Interpretation* The predominance of the grainstone texture suggests high-energy environments. The abundance of green algae points to a depositional setting within the photic zone, from a lagoonal environment to shallow shoals (Mamet 1991). However, the association of green algae, *Tubiphytes* sp. and phylloid algae strongly suggest open-marine conditions (e.g. Mamet 1991; Dawson and Racey 1993). This interpretation is supported by the occurrence of suspension feeders (bryozoans, brachiopods and echinoderms) which require well-oxygenated waters and normal salinity (Wilson 1975).

### Coated-grain grainstone

**Fig. 8:**
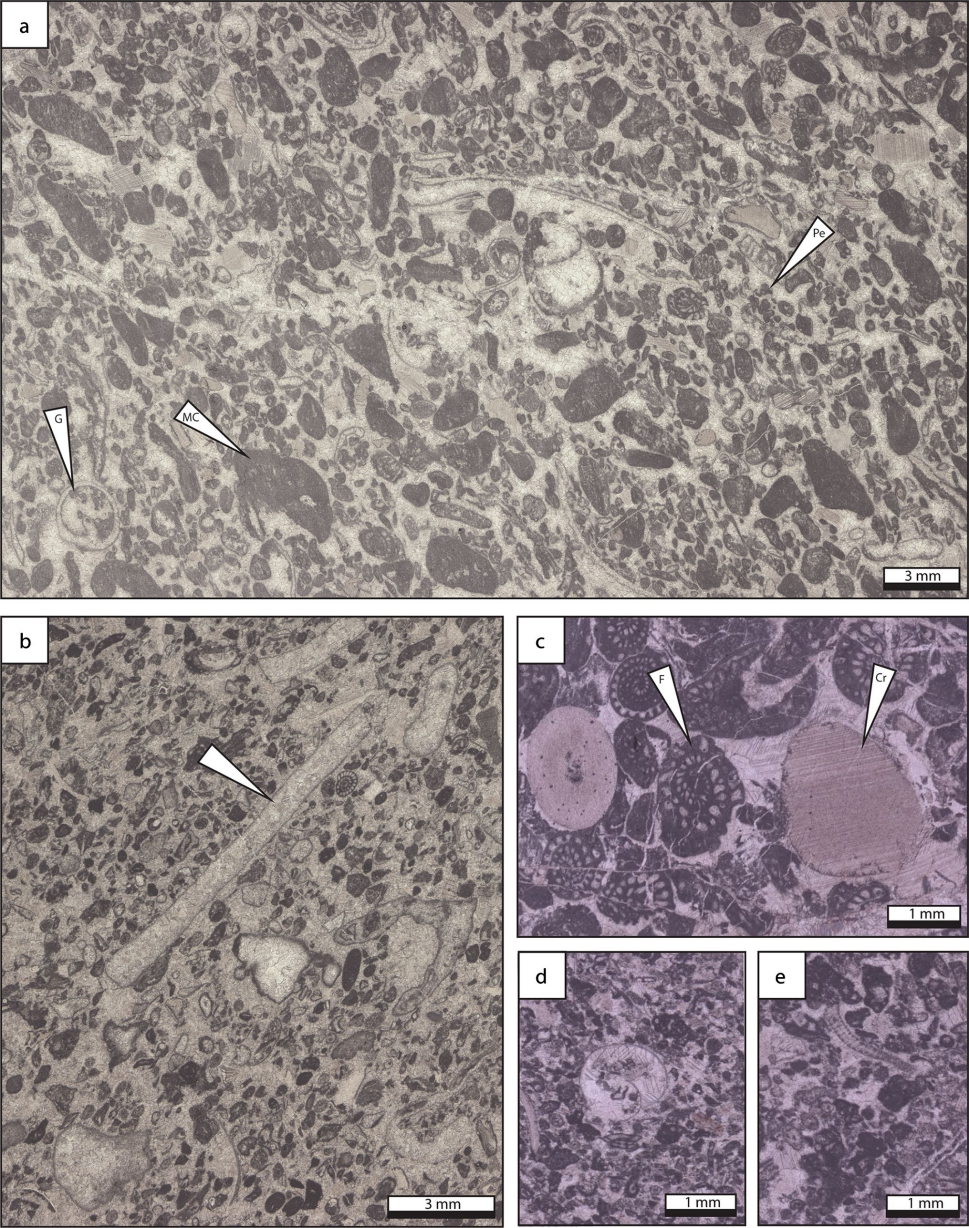
Coated grain grainstone lithofacies type which represents an updip shallow-water, high energy paleoenvironment. **a** Grainstone composed of molluscan bioclasts, including small gastropods, and darkened micritized and coated bioclasts and peloids. **b** Fine-grained peloidal grainstone matrix with sparse coarser-grained mollusc and algal fragments. **c** Coarse-grained fusuline-crinoid grainstone. **d** Fine-grained peloidal grainstone with coarser-grained gastropod shell fragment. **e** Peloidal–bioclastic grainstone with sparse *Beresella* algal fragments. *Cr* crinoid, *F* foraminifera, *G* gastropod, *MC* micritized grain, *Pe* peloid

*Description* Coated-grain grainstone is characterized by abundant subspherical to ellipsoid cortoids, up to 50%, ranging in size from 0.2 to 2 mm (Fig. 8). The coated biogenic components include phylloid algae, echinoderms, fenestrate bryozoans, gastropods, bivalves, *Tubiphytes* sp., the green algae *Epimastopora* sp. and *Beresella* sp., foraminifera (*Schwageriniformis*, *Rauserites*) and brachiopods. These bioclasts are associated with peloids and scarce mud clasts (< 2 mm in diameter). The cortex of cortoids consists of a thin non-laminated micrite envelope. The lamina surface is smooth. Radiaxial-fibrous, blocky and drusy calcite cements are common.

*Interpretation* Cortoids resulted from destructive micritization process linked to the activity of microboring organisms. These microborers, light dependent, are commonly interpreted as an indicator of shallow-marine conditions (< 20 m; Swinchatt 1969; Lees and Miller 1985; Aretz and Herbig 2008). Additionally, the occurrence of green algae points to a setting within the photic zone. In Spain, *Epimastopora*, associated with other dasycladaceans and oncoids, is abundant in outer-shelf facies and occurs commonly in shallow and high-energy environments (Della Porta et al. 2002). Therefore, shallow and agitated waters, located above the FWWB in the photic zone, such as those prevailing in shoals (e.g. Kumpan et al. 2014; Udchachon et al. 2014; Erfani et al. 2016), are inferred.

### Bioclastic packstone, grainstone, floatstone, rudstone

**Fig. 9:**
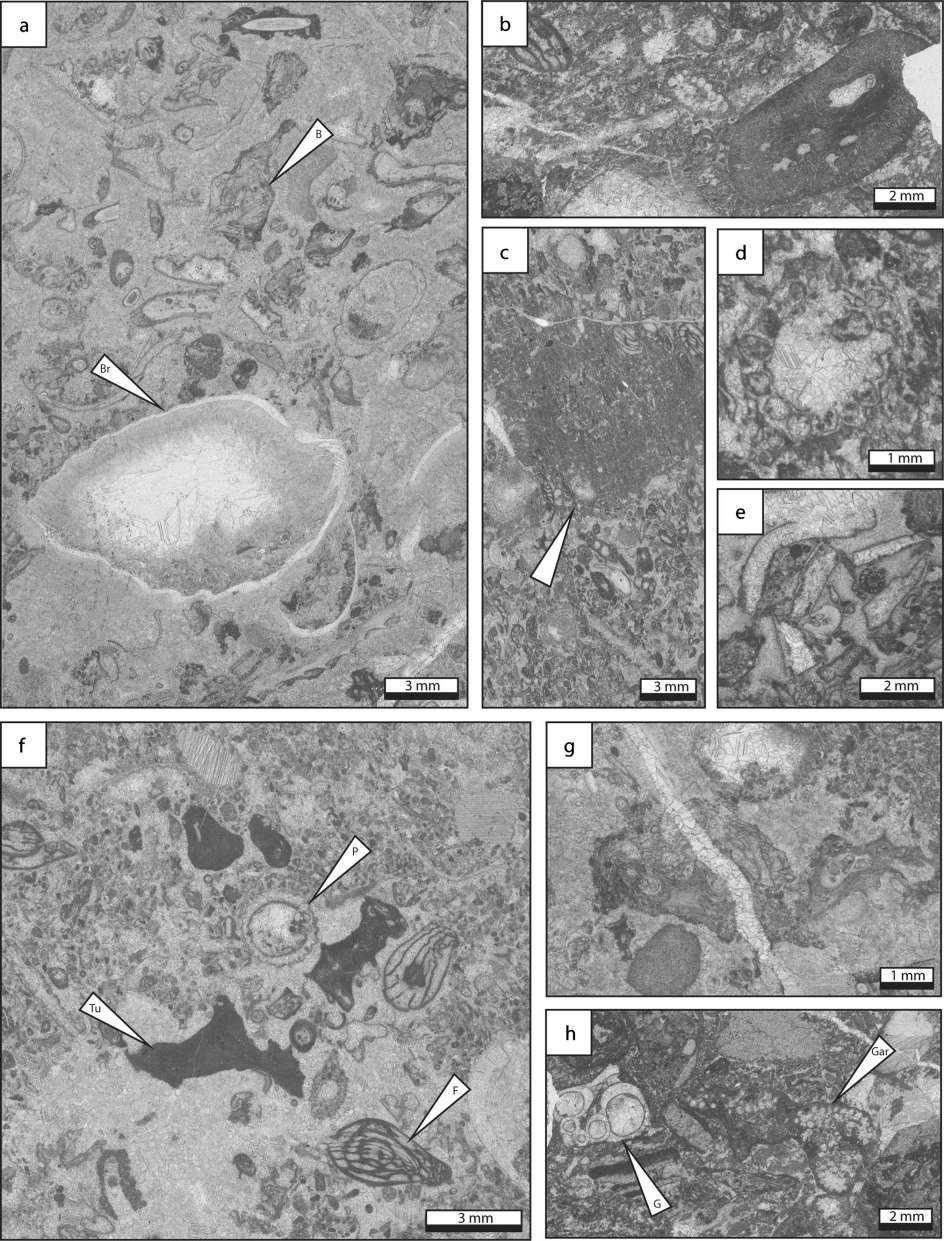
Bioclastic grainstone, packstone, rudstone and floatstone lithofacies type. The lithofacies is composed of diverse bioclasts, including **a** bryozoans, brachiopods, molluscs, **b**, **f** *Tubiphytes*, **c** mud-filled burrows in foraminifer-rich packstone matrix, **d** *Gyroporella* sp., **e** phylloid algae fragments, foraminifera, *Pseudogyroporella* sp., **g** fistuliporid bryozoans, **h** *Garwoodia* sp. and gastropods. *B* bryozoan, *Br* brachiopod, *F* foraminifera, *G* gastropod, *Gar* *Garwoodia* sp., *P* *Pseudogyroporella*, *Tu* *Tubiphytes* sp

*Description* Bioclastic packstone, grainstone, floatstone and rudstone, locally burrowed, are characterized by the abundance of diverse angular fragments of skeletal components, poorly sorted, ranging in size from 0.1 to 5 mm (Fig. 9). Most of the biogenic components consist of fenestrate and fistuloporid bryozoans, *Tubiphytes* sp., foraminifera (*Schwageriniformis*, staffellids, schwagerinids, *Rauserites, Ozawainella, Schubertella, Fusiella*) and brachiopods associated to solitary and colonial rugose corals, echinoderms, bivalves, phylloid algae (unspecified), green algae (*Beresella* sp., *Pseudogyroporella* sp., *Gyroporella* sp., *Epimastoporella* sp., *Epimastopora* sp., *Diplopora* sp.*?*), calcimicrobes (*Archeolithoporella* sp., *Garwoodia* sp.), gastropods and worm tubes (*Thartharella* sp.). Locally, some intervals consist of densely packed phylloid algal thalli, colonial and solitary corals or brachiopod shells. Scarce peloids, mud clasts and coated grains occur. Intergranular porosity is infilled by fibrous, radiaxial-fibrous, drusy, blocky and locally dog tooth cements.

*Interpretation* Autochthonous bioclastic packstone, grainstone, floatstone and rudstone are characterized by poorly sorted whole fossils and fragments, which suggest in situ components. The occurrence of burrows suggests shallow lagoon with open marine circulation, deep shelf or mid and outer ramp settings (Henderson et al. 1999; Flügel 2010). However, the association of dasyclad green algae, phylloid algae and calcimicrobes points to a depositional setting within the photic zone (Mamet 1991). In addition, the occurrence of suspension feeders (e.g. bryozoans, brachiopods and corals) which require well-oxygenated waters and normal salinity (Wilson 1975) suggest open marine depositional environment. All these elements allow to constrain the depositional environment to a reef flank setting (Flügel 2010), in a shallow subtidal environment, located in the photic zone.

Allochthonous bioclastic packstone, grainstone, floatstone and rudstone is characterized by abraded fossil fragments, associated with mud clasts. Grains occur in chaotic order or present grading texture. Skeletal fragments consist of reef-derived organisms, likely transported down by turbidites, deposited in forereef position, reef slope, toe-of-slope, and basinal settings (Flügel 2010).

### Microbial boundstone

**Fig. 10:**
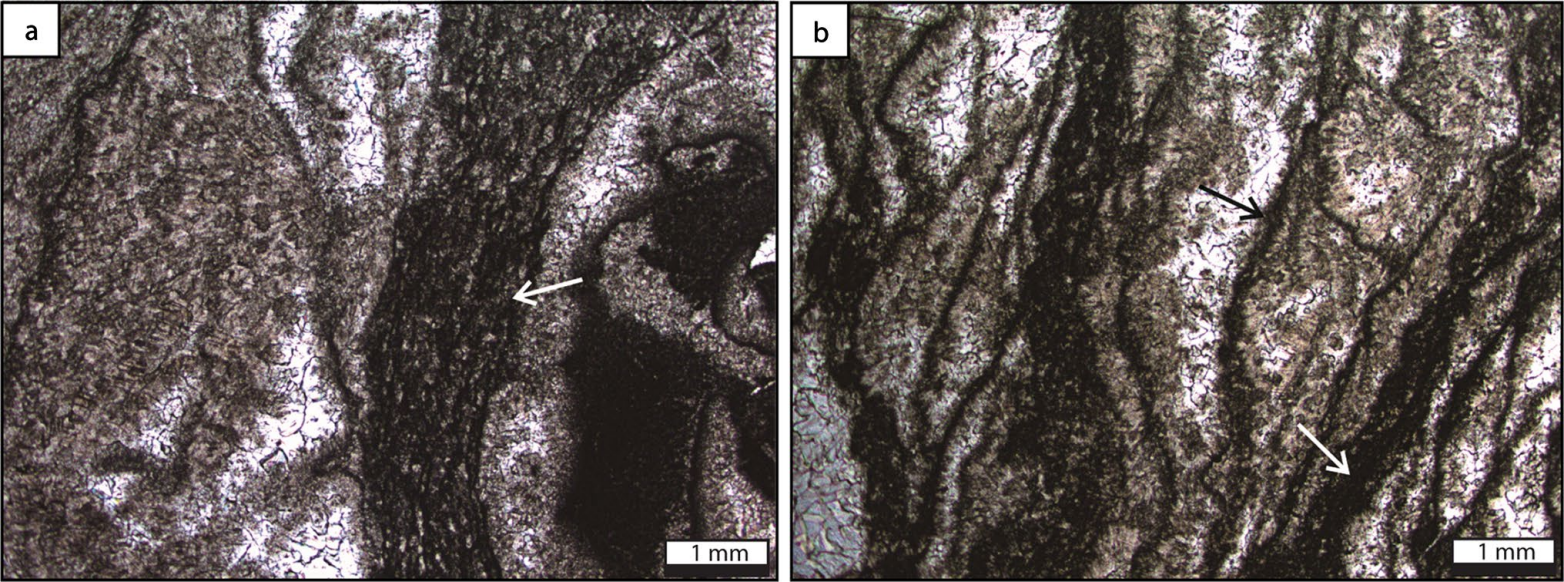
Microbial boundstone lithofacies type, built by undetermined algal-like microbial fabrics. **a** Encrusting form (white arrow), with the fuzzy structure encrusting the thrombolitic clasts. **b** Laminar (white arrow) and grid-shaped (black arrow) microbial microfabrics. From Huang et al. (2019)

*Description* Microbial boundstone is characterized by stromatolitic internal structures and wrinkle structures on the weathered surface, which is composed of abundant marine cement and encrusting microbial micrite. Three microscopic structures of the microbial boundstone have been identified, including encrusting, laminar, and grid-shaped microbial fabrics (Fig. 10, Huang et al. 2019). Abundant microbial carbonate (e.g. thrombolitic textures, microstromatolites, and microbial ooids) and calcimicrobes (e.g. *Girvanella*, *Ortonella*, and *Wetheredella*-like) occur in the deposits between microbial boundstone and the coral *Ivanovia* (Huang et al. 2019). Subordinate contributors include corals, crinoids, bryozoans, algae, and sponges.

*Interpretation *Microbial boundstone, in association with the coral *Ivanovia*, form the Zhongxinzhai mound (Houchang town, Guizhou). The association of microbial boundstone with numerous calcimicrobes, algae, corals and bryozoans, may indicate that the microbial boundstone was formed in the euphotic zone, with normal salinity condition (Gong et al. 2007a; Zhang et al. 2009; Huang et al. 2019). *Ivanovia* encrusts hard substrates and favors shallow, clear, high-energy environments (Zhang et al. 2009), which display an alternating encrusting growth pattern with the microbial boundstone, suggesting a shallow and clear water.

### Phylloid green algal boundstone

**Fig. 11:**
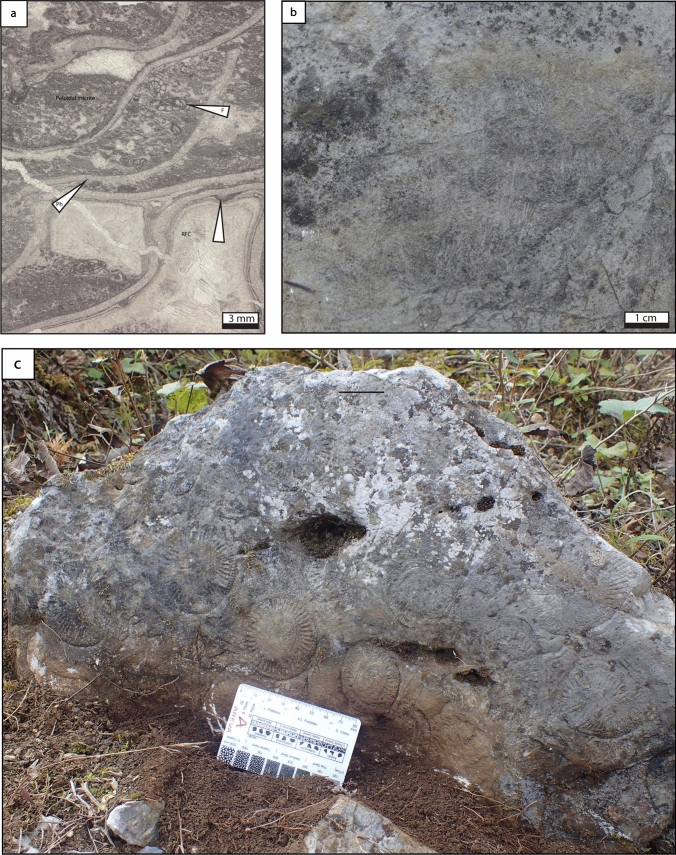
Phylloid algal and coral boundstone lithofacies types. **a** Phylloid algal boundstone. Pores are filled by fine-grained peloidal packstone and marine radiaxial calcite cement. Algal thalli are draped by conspicuous marine cement or encrusted by microbialite and *Tubiphytes* prior to the precipitation of marine cement in interstitial space. Matrix contains common fusuline foraminifera. **b** *Ivanovia* coral. **c** Branching rugose corals (*Fomitchevella*) formed large colonies (> 50 cm), preserved in their original up-right position. *F* foraminifera, *Ph* phylloid algae, *RFC* radiaxial fibrous cement

*Description* Phylloid algae form a dense framework with shelter cavities filled by marine cement (radiaxial-fibrous, drusy and blocky) and peloidal micrite (Fig. 11a). The undulate leaf-like algal thalli are commonly recrystallized. Additional organisms include foraminifera (*Schwageriniformis*), echinoderms, the green algae *Epimastopora* sp. and *Pseudogyroporella* sp., bryozoans, *Tubiphytes* sp., worm tubes (*Thartharella* sp.) and bivalves along with scarce mud clasts. The peloidal matrix is composed of dense, clotted or partly laminated micrite.

*Interpretation* Phylloid algae are commonly reported as growing in shallow waters, under moderate energy conditions, within the photic zone (e.g. Beauchamp et al. 1989; Wahlman 2002; Gong et al. 2007a, b). This interpretation is supported by the occurrence of dasyclad green algae and *Tubiphytes* sp.*,* commonly growing in shallow-waters (Mamet 1991). The high diversity biotic association including brachiopods, bryozoans, and foraminifera, attest for open marine conditions and well-oxygenated waters (Oertli 1964; Wilson 1975). Phylloid algal mounds are extensively documented in the literature as growing in relatively shallow subtidal settings (e.g. Toomey et al. 1977; Soreghan and Giles 1999; Samankassou and West 2002).

### Ivanovia cf. manchurica boundstone

*Description* In Houchang, the encrusting *Ivanovia* cf. *manchurica* (Fig. 11b) is considered as an aphroid coral (e.g. Zhang et al. 2009; Gong et al. 2012). Within the Zhongxinzhai mound, *Ivanovia* cf. *manchurica* measures about 1.2 cm in diameter. *Ivanovia* boundstone forms massive-shaped domes, reaching 2 m lateral extension and 0.3–0.7 m thickness. The matrix consists of bioclastic grainstone, mainly composed of crinoid stems.

*Interpretation* The grainstone texture suggests agitated waters. In addition, *Ivanovia* are common in intertidal and shallow subtidal environments, in warm and clear marine environment within the photic zone (Taylor and Wilson 2003; Zhang et al. 2009). Therefore, the *Ivanovia* cf *manchurica* boundstone formed likely in shallow-water and high energy depositional environment, above the FWWB.

### Branching coral boundstone

*Description* Branching colonial corals (*Fomitchevella* sp.) form a distinct framework, occurring commonly in their original up-right position. Branching corallites reach a maximum of 7 cm in diameter and 60 cm height (Fig. 11c). The matrix consists of micrite, including fragments of algae (undetermined), brachiopods (*Choristites*, *Martinia*) and crinoids, and few fusulines (*Triticites*, *Schwagerina,* Gong et al. 2004; Gong et al. 2012).

*Interpretation *Chappell (1980) suggested that branching corals appear as moderately light-dependent, living in calm waters under high sediment influx, in subtidal settings. Scrutton (1999) suggested that fasciculate growth forms were favored by rapid sedimentation rates and less frequent scouring event. The enclosing sediments composed of micrite with scarce skeletal debris (algae, crinoids, brachiopods) confirm low-energy conditions (Scrutton 1999). Therefore, the branching coral boundstone is interpreted as growing in a low energy depositional environment, below the FWWB.

### Burrowed bioclastic wackestone

**Fig. 12:**
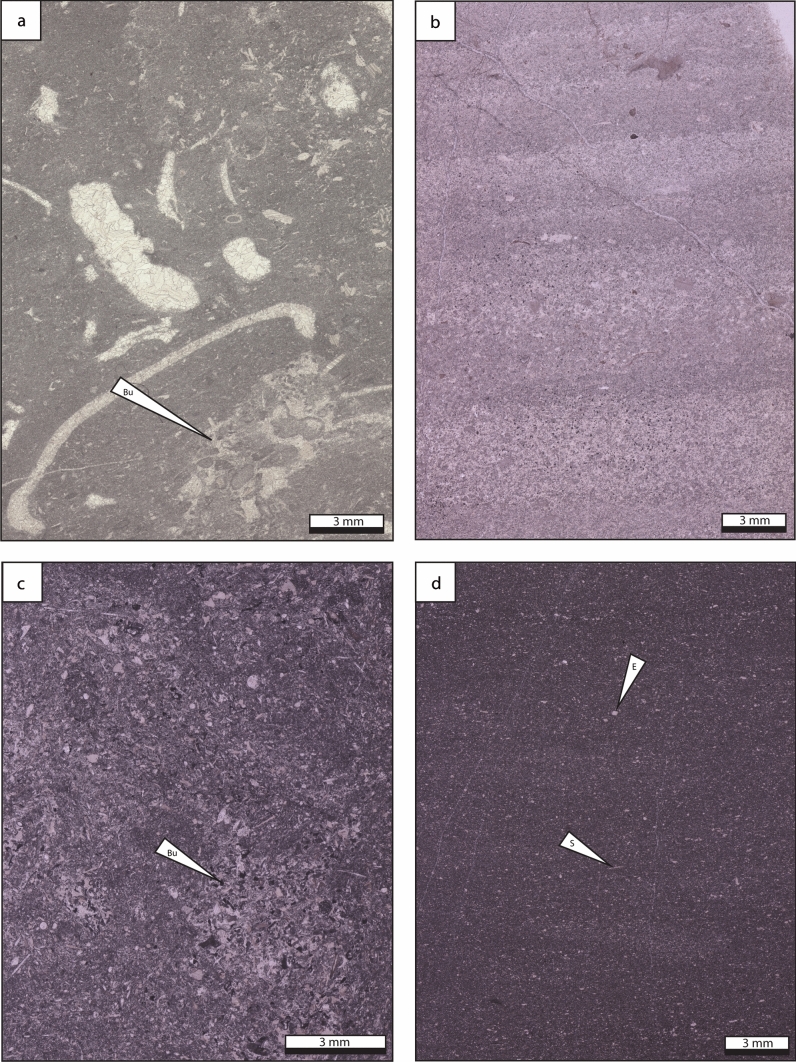
Burrowed bioclastic wackestone, microbioclastic peloidal packstone–grainstone and fine-grained burrowed wackestone–packstone lithofacies types, which represent a deep-water, low energy paleoenvironment. **a** Burrowed bioclastic wackestone. Biogenic components consist of foraminifera, debris of echinoderms and phylloid algae. Locally, scarce peloids occur. **b** Laminated microbioclastic peloidal packstone and grainstone with fine peloids and exhibiting mm-scale layering. **c** Fine-grained burrowed wackestone and packstone composed of small-sized fossil fragments, scattered within a dark and strongly burrowed matrix. Burrows are infilled with microbioclastic peloidal packstone and grainstone sediments. **d** Fine-grained burrowed wackestone. Matrix consist of dark grey micrite. Bioclasts include small echinoderm fragments and spicule sponges. *Bu* burrow, *E* echinoderm, *S* spicule sponge

*Description* Burrowed bioclastic wackestone is composed of bioclast fragments which include phylloid algae, bryozoans, foraminifera, echinoderms, bivalves, brachiopod spines, *Tubiphytes* sp. and *Donezella* sp. (Fig. 12a). Peloids, mud clasts and coated grains occur scarcely. Vugs are filled by drusy and blocky calcite cements.

*Interpretation* The burrowed bioclastic wackestone could have originated as either lagoonal deposits or as basinal background sedimentation, deposited below the FWWB. The occurrence of burrows suggests shallow lagoon with open marine circulation, deep shelf or mid and outer ramp settings (Henderson et al. 1999; Flügel 2010). The lack of light-dependent dasyclad algae allows to restrain the depositional environment to the dysphotic zone (Mamet 1991). Therefore, the origin of the burrowed skeletal wackestone is interpreted as in situ sedimentation, deposited in a low-energy environment below the FWWB and certainly below the SWB (e.g. Casier et al. 2004).

### Microbioclastic peloidal packstone and grainstone.

*Description* Microbioclastic peloidal packstone and grainstone is composed of detrital silt-sized calcite particles (2–63 μm), including peloids, litho- and bioclast fragments (Fig. 12b). The biogenic components consist of disarticulated crinoids, shell debris and scarce ostracods. Millimeter-scale ripple cross-lamination and laminae occur.

*Interpretation* Microbioclastic peloidal packstone and grainstone are produced by abrasion and bioerosion processes. The low-angle cross-stratification suggests oscillatory flows, likely induced by storms, or turbidity currents (e.g. Kim and Lee 1996; Seguret et al. 2001; Woo and Chough 2007; Chen et al. 2011; Merhabi et al. 2015). Microbioclastic peloidal packstone and grainstone occur commonly in toe-of-slope, outer ramp and basinal environments (Flügel 2010).

### Fine-grained burrowed wackestone and packstone

*Description* Dark-colored fine-grained burrowed wackestone and packstone are composed of microfossils, including sponge spicules, shell debris and echinoderm fragments (Fig. 12c, d). Bioclasts are scattered within a dense and strongly burrowed mud matrix. Locally, skeletal fragments are densely packed or sparsely distributed due to the intense burrowing.

*Interpretation* The skeletal assemblage, composed of fine-grained benthic biodetritus including spicule sponges, are common in deep-marine environments, both in slope and basinal settings (Wiedenmayer 1980; Jach 2001). The abundance of burrows supports this statement, occurring commonly in the deep shelf, mid and outer ramp settings (Henderson et al. 1999; Flügel 2010). Therefore, the fine-grained burrowed wackestone and packstone were deposited in outer ramp, deep shelf, and basinal settings with aerobic to possibly dysaerobic bottom conditions (Flügel 2010).

## Lithofacies successions

### Shallow-water section (section 1)

**Fig. 13:**
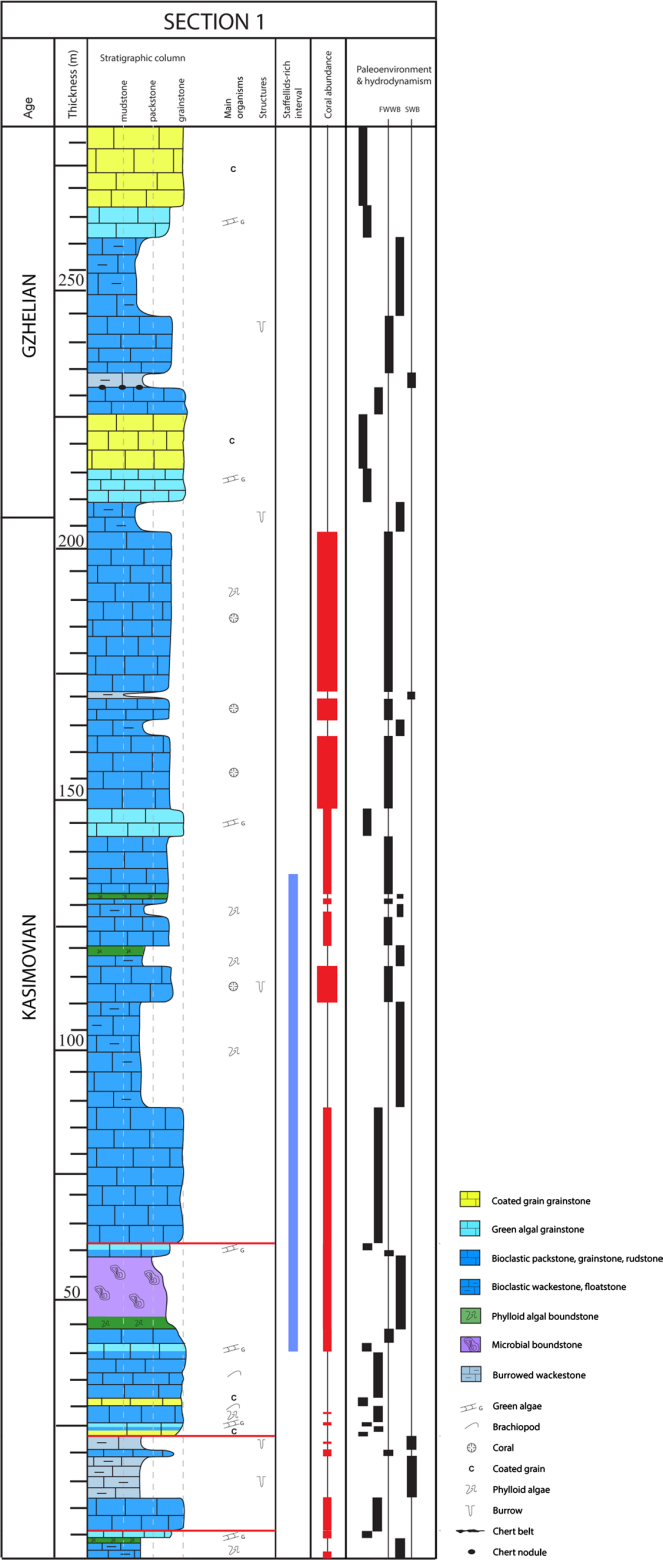
Lithofacies successions and paleoenvironments, from section 1 (Zongxinzhai), showing distributions of corals and staffellid foraminifers. The abundance of staffellids in the lower section indicate shallow-water, possibly restricted marine paleoenvironments, and the overlying coral-rich facies indicates a significant sea level rise and a change to a deeper-water shelf margin setting

**Fig. 14:**
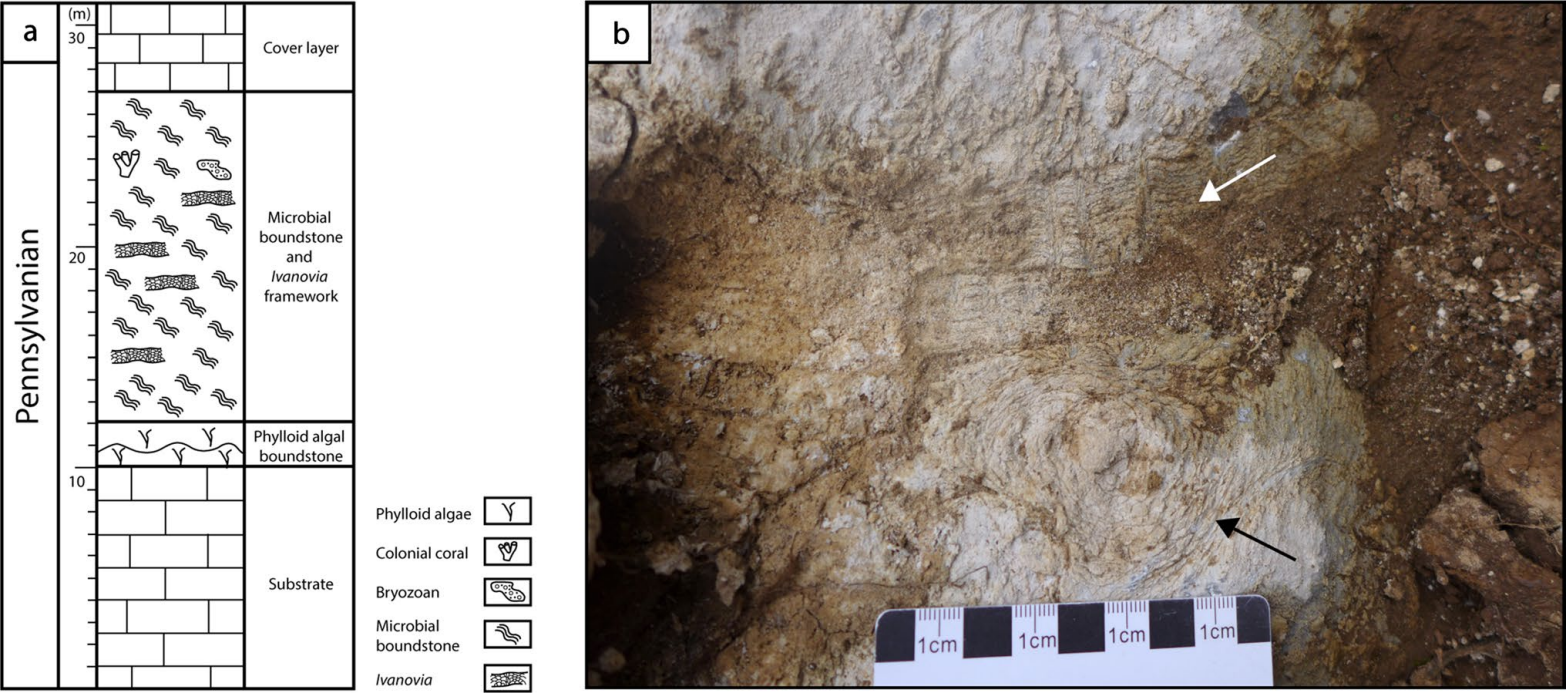
Zhongxinzhai microbial mound. **a** Stratigraphic column of the Zhongxinzhai mound showing the composition and distribution of reef-building organisms. **b** Photograph of the mound core. The coral *Ivanovia* (white arrow) grows on the microbial boundstone (black arrow). Modified from Huang et al. (2019)

The lithofacies succession of section 1 suggests shallow-water depositional environments (Fig. 13). The rock succession is dominated by thick limestone beds (> 2 m), dated as Kasimovian to Gzhelian in age. Lithofacies include coated grain grainstone, green algal grainstone, bioclastic packstone, grainstone and rudstone, bioclastic wackestone and floatstone, phylloid algal boundstone, microbial boundstone and burrowed wackestone. From 44 to 59 m, a microbial mound (15 m high, 200 m wide, Fig. 14) was identified, with the secondary contribution of phylloid algae and the coral *Ivanovia*. The substrate consists of bioclastic wackestone and packstone composed of various organisms including microbialite debris, crinoids, foraminifera, bryozoans, brachiopods, gastropods and algae, along with peloids and coated grains. The base of the mound is characterized by phylloid algal bafflestone (2 m thick and 8 m wide) and the core is built by microbial boundstone and the coral *Ivanovia*. The grain assemblage suggests a subtidal paleoenvironment, deposited in shallow-waters, within the photic zone (Huang et al. 2019).

### Deep-water section (section 2)

**Fig. 15:**
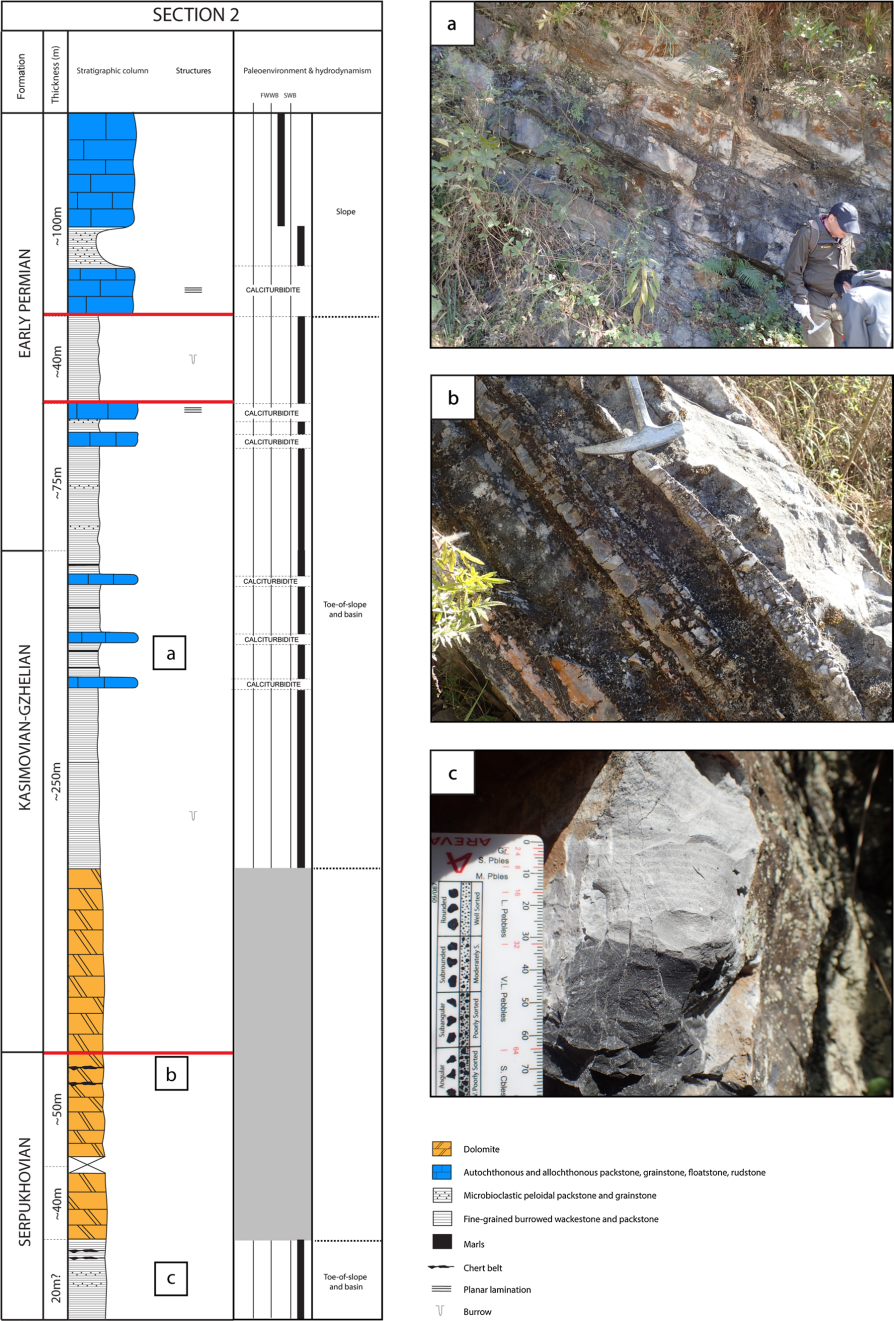
Lithofacies successions and paleoenvironments, from section 2 (Brickyard). During the latest Mississippian–earliest Pennsylvanian, lithofacies are commonly dolomitized, inhibiting the original texture to be identified. During this time-interval there is a probable unconformity, with the lack of Bashkirian deposits. During the Kasimovian–Early Permian (Asselian), lithofacies suggest toe-of-slope and basinal settings, accumulated in the Luodian intraplatform Basin. During the late Early Permian (Sakmarian), lithofacies indicate likely reef slope settings, which suggests a regression. **a** Field photograph of alternating of well-bedded fine-grained burrowed wackestone and packstone, thin marl and scarce allochthonous packstone and grainstone layers (< 80 cm thick). **b** Field photograph of chert belts. **c** Field photograph of microbioclastic peloidal packstone and grainstone turbidite bed unit displaying small-scale ripple cross-lamination. The underlying layer is composed of fine-grained burrowed wackestone and packstone

The lithofacies succession of section 2 is dominated by deep-water deposits (Fig. 15) ranging in age from the Serpukhovian to Early Permian. The Serpukhovian is characterized by the deposition of chert belts (Fig. 15b), fine-grained burrowed wackestone and packstone, and microbioclastic peloidal packstone and grainstone, exhibiting commonly millimeter-scale ripple cross-lamination and laminae (Fig. 15c). During the latest Mississippian–earliest Pennsylvanian, the sediment succession was dolomitized, likely due to the global sea-level fall (Ross and Ross 1987; Rygel et al. 2008). Then, from the Kasimovian?—Early Permian (Asselian), the sedimentary succession is dominated by fine-grained burrowed wackestone and packstone, microbioclastic peloidal packstone and grainstone (< 1 m thick), associated with thin marls (few centimeters) and allochthonous bioclastic packstone, grainstone and rudstone beds, commonly laminated (planar lamination; Fig. 15a). During the late Early Permian (Sakmarian), lithofacies are composed of in situ bioclastic packstone, grainstone and rudstone layers.

### Bianping coral reef section

**Fig. 16:**
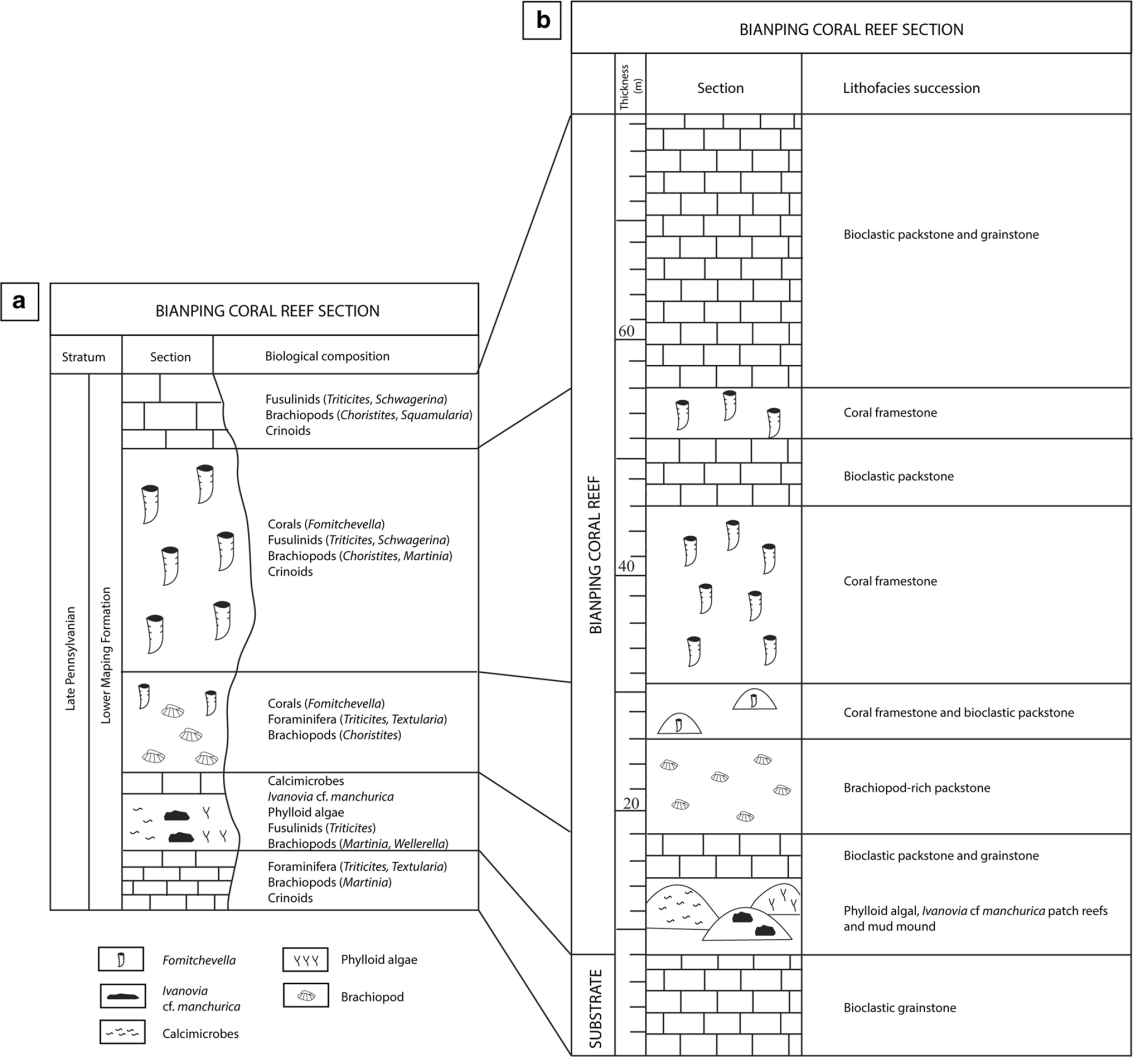
Bianping coral reef sections. **a** A summary of the biological composition of the Bianping coral reef, modified from Gong et al. (2012). **b** Bianping coral reef section showing the lithofacies succession, modified from Gong et al. (2004)

The Bianping coral reef section (Fig. 16) was described by Gong et al. (2004). The reef substrate is composed of bioclastic grainstone, including foraminifera (*Triticites*, *Textularia*), brachiopods (*Martinia*) and crinoids. The lower part of the reef (10 m thick) consists of three patch-reefs, built by phylloid algae, calcimicrobes and the coral *Ivanovia* cf *manchurica*, respectively. These patch-reefs are overlain by bioclastic packstone and grainstone, prior to be capped by a brachiopod-rich packstone layer (8 m thick), dominated by *Choristites* brachiopods, foraminifera (*Triticites*, *Textularia*) and corals (*Fomitchevella*). Above, the coral reef core (37 m thick) is dominated by the *Fomitchevella* branching colonial corals. Between the coral framework occur bioclast packstone deposits, composed of fusulines (*Triticites*, *Schwagerina*), brachiopods (*Choristites*, *Martinia*) and crinoids. The basal part of the reef (5 m) is composed of isolated coral patch-reef frameworks with interstitial packstones of densely packed bioclasts of crinoids, fusulines and algal debris. Above, branching corals built a large and massive framework (15 m thick), capped by a bioclastic packstone layer (13 m thick) composed of fusulines and brachiopods. The upper 4 m of the reef is composed of massive coral framestone. Between the coral framework occur bioclastic packstone deposits. Finally, the reef cover consists of bioclastic packstone and grainstone, including fusulines (*Triticites*, *Schwagerina*), brachiopods (*Choristites*, *Squamularia*) and crinoids.

## Interpretations

### Depositional environment of the Bianping coral reef

The depositional environment of the Bianping coral reef is currently not well constrained. However, its lithofacies succession provides precious information about the depositional environment (Fig. 16). The reef substrate, composed of bioclastic grainstone, including foraminifera brachiopods and crinoids, is indicative of a high-energy depositional environment, located above the FWWB. Then, the biotic assemblage of the lower part of the reef (calcimicrobes, *Ivanovia* cf. *manchurica* and phylloid algae) suggests shallow subtidal settings, deposited around the FWWB within the photic zone. Conversely, the reef core composed of branching coral framestone (*Fomitchevella,* Fig. 11c) and bioclastic packstone indicates a slightly lower-energy and deeper environment, deposited below the FWWB. The reef cover, composed of bioclastic packstone and grainstone suggestive of a shallower environment, deposited in more agitated waters, around the FWWB.

### Bianping coral reef: a world exception

At tropical to subtropical latitudes, Bashkirian–Moscovian shelf to shelf-margin bionconstructions were dominated by mounds and banks constructed by calcareous algae (e.g. West 1988), *Chaetetes* sponges (e.g. Connolly et al. 1989; Hamilton 2014) and microbialites (e.g. Samankassou 2001; Della Porta et al. 2003; Bahamonde et al. 2007). In the Late Moscovian, the composition of tropical shallow-water organic buildups changed significantly with the radiation of erect phylloid algae, which became the most dominant reef-builder throughout Late Pennsylvanian and earliest Permian (e.g. Heckel and Cocke 1969; Toomey 1991; Gong et al. 2007a, b; Samankassou and West 2002; Wahlman 2002). In slightly deeper water settings, small buildups were constructed by erect bryozoans, crinoids, and calcareous sponges that were encrusted by laminar red algae (e.g. *Archaeolithophyllum*), fistuliporid bryozoans and *Tubiphytes* (e.g. Toomey 1979; Schatzinger 1983; Wahlman 2002).

However, scarce Pennsylvanian coralliferous bioconstructions were also reported worldwide (Table 3).

1. In Oklahoma (US), Late Viséan–Early Bashkirian *Petalaxis* coral patch reefs were reported in shallow subtital to intertidal settings (Sutherland and Henry 1977).
2. In Kazakhstan, Middle Viséan–Early Bashkirian coral–crinoid–bryozoan–algal–*Tubiphytes* reefs were discovered at the platform margin (Cook et al. 1994).
3. In Japan, the Akiyoshi Limestone Group represents a large reef complex that developed on a volcanic seamount. During the Viséan–Early Bashkirian, the initiation of the Akiyoshi reef complex was led by the proliferation of corals, bryozoans and crinoids (Ota 1968; Nakazawa 1997). During the Bashkirian–Moscovian, the Akiyoshi reef cores included chaetetids, calcareous algae, bryozoans, and tabulate and rugose corals (Nagai 1985; Sugiyama and Nagai 1990, 1994). Sugiyama and Nagai (1990, 1994) interpreted the Akiyoshi buildup as a high energy walled-reef complex, analogous to modern coral oceanic atolls.
4. In Ukraine (Donets Basin), Bashkirian and Early Moscovian coral and coral-chaetetid biostromes (< 6 m thick) were reported in shallow waters (Ogar 2012). In detail, the Pashenna bioherm (> 3 m thick, section) is dominated by chaetetids and corals (*Ivanovia*). The Holubivka bioherm (> 3 m thick) is composed of brachiopods, chaetetids, solitary rugose corals (*Yuanophylloides*), massive colonial corals (*Petalaxis*) and stromatolites. The Karahuz bioherm (6 m thick) includes microbialites, foraminifera, worm tubes, crinoids, solitary rugose corals (*Axolithophyllum*, *Yuanophylloides* and *Monophyllum)*, tabulate corals (*Cladochonus*), and bryozoans (6 m thick). The Maryivka biostromes are composed of corals, calcareous algae, crinoids, brachiopods and foraminifera.
5. In Spain, Dingle et al. (1993) described Bashkirian–Moscovian coral-*Chaetetes* buildups (few centimeters thick), dominated by *Chaetetes*, solitary rugose corals (*Caninia*) and tabulate corals (*Multithecopora*). These buildups developed in shallow waters, high-energy environments (packstone–grainstone facies). Bahamonde et al. (2015, 2017) reported Moscovian coral bioherms and biostromes (10’s-cm-thick) composed of undetermined branching rugose coral colonies associated with cerioid colonies, solitary rugose corals, auloporid corals and *Chaetetes.* These bioconstructions were deposited in moderate-energy and relatively shallow-water environments, below the FWWB.
6. In Nevada (US), Moscovian *Chaetetes* reefs were discovered. The main reef-builders are *Chaetetes* associated to fusulines, algae (*Ivanovia*, *Donezella*, *Dvinella*), rugose corals (*Caninia*, *Amandophyllum*, *Caniniostrotion*, *Tschussovskenia*), tabulate corals (*Syringopora*, *Multithecopora*) and bryozoans (Wilson 1963; Nelson and Langenheim 1980; Gong et al. 2012).
7. In the Moscow Basin, Late Moscovian coral biostromes and bioherms were described, dominated by rugose corals (*Ivanovia*, *Petalaxis*) and chaetetids (Podolskian and Myachkovian Horizons; Ogar 2012).
8. In Austria–Italy (Carnic Alps), Kasimovian coral mounds (50 cm thick) were identified (Samankassou 2003), composed of auloporid corals (*Multithecopora syrinx*), associated to sponge spicules (?), worm tube structures, shell fragments, foraminifera, ostracods, and chaetetid sponges. Coral mounds grew in a low-energy depositional environment.

**Table 3:** A synthesis of Pennsylvanian coralliferous bioconstructions

Country	Age	Reef	Reef-buiders	Thickness	Environment	References
Southern China	Gzhelian	Coral reef	Branching rugose corals (*Fomitchevella*), phylloid algae, *Ivanovia* cf. *manchurica*, *Antheria*, microbialites, brachiopods, foraminifera, crinoids, algae, bryozoans and *Tubiphytes*	80–100 m thick	Low-energy environment	9
Austria–Italy, Carnic Alps	Kasimovian	Coral mounds	Auloporid corals (*Multithecopora syrinx*), sponge spicules (?), worm tube structures, shell fragments, foraminifera, ostracods, and chaetetid sponges	Few cm to 50 cm thick	Low-energy environment	8
Russia, Moscow Basin	Late Moscovian	Coral biostromes and bioherms	Rugose corals (*Ivanovia*, *Petalaxis*) and chaetetids	X	x	7
US, Nevada	Moscovian	*Chaetetes* reefs	*Chaetetes*, fusulinids, algae (*Ivanovia*, *Donezella*, *Dvinella*), rugose corals (*Caninia*, *Amandophyllum*, *Caniniostrotion*, *Tschussovskenia*), tabulate corals (*Syringopora*, *Multithecopora*) and bryozoans	X	X	6
Spain	Moscovian	Coral bioherms and biostromes	Undetermined branching rugose coral, cerioid corals, solitary rugose corals, auloporid corals and *Chaetetes*	10′s cm thick	Moderate energy environment, relatively shallow waters, below the FWWB	5
	Bashkirian–Moscovian	Coral-*Chaetetes* buildups	*Chaetetes*, solitary rugose corals (*Caninia*) and tabulate corals (*Multithecopora*)	Few cm thick	Shallow-water and high energy environment	
Ukraine, Donets Basin	Bashkirian–Early Moscovian	Coral and coral-*Chaetetes* biostromes	Pashenna bioherm: chaetetids and corals (*Ivanovia*)	> 3 m thick	Shallow waters	4
			Holubivka bioherm: brachiopods, chaetetids, solitary rugose corals (*Yuanophylloides*), massive colonial corals (*Petalaxis*) and stromatolites	> 3 m thick		
			The Karahuz bioherm: microbialites, foraminifera, worm tubes, crinoids, solitary rugose corals (*Axolithophyllum*, *Yuanophylloides, Monophyllum)*, tabulate corals (*Cladochonus*), and bryozoans	6 m thick		
			Maryivka biostromes: corals associated to calcareous algae, crinoids, brachiopods and foraminifera	X		
Japan, Akiyoshi Limestone Group	Viséan–Moscovian	Metazoan atoll	Chaetetids, calcareous algae, bryozoans, tabulate and rugose corals, crinoids	Large size	High energy walled-reef complex	3
Kazakhstan	Middle Viséan–Early Bashkirian	Coral–crinoid–bryozoan–algal–*Tubiphytes* reefs	Coral–crinoid–bryozoan–algal–*Tubiphytes*	X	Platform margin	2
US, Oklahoma	Late Viséan–Early Bashkirian	Coral patch reefs	*Petalaxis* corals	Small size	Shallow subtidal to intertital	1

1—Oklahoma (US): Sutherland and Henry (1977); 2—Kazakhstan: Cook et al. (1994); 3—Akiyoshi Limestone Group (Japan): Ota (1968), Nagai (1985), Sugiyama and Nagai (1990, 1994), Nakazawa (1997); 4—Donets Basin (Ukraine): Ogar (2012); 5—Spain: Dingle et al. (1993), Wahlman (2002), Bahamonde et al. (2015, 2017); 6—Nevada (US): Wilson (1963), Nelson and Langenheim (1980), Gong et al. (2012); 7—Moscow Basin: Ogar (2012); 8—Austria–Italy: Samankassou (2003); 9—Southern China: Zhang et al. (2010), Gong et al. (2012)

All of these Pennsylvanian coralliferous bioconstructions were dated from Bashkirian–Kasimovian, with a Gzhelian “reefless lag time”, and grew in environments ranging from shallow-waters (above the FWWB) to the low-energy environment (below the FWWB). The reef-size remains small, measuring 10’s centimeter to meter thick (< 6 m), except in Japan (large atoll). The reefal assemblages were commonly composed of corals, chaetetids*,* calcareous algae, foraminifera, crinoids, molluscs, bryozoans, associated locally to worm tubes, microbialites, stromatolites and *Tubiphytes*.

Consequently, the Bianping reef in Houchang, southern China (Zhang et al. 2010; Gong et al. 2012), adds to the current knowledge a new Late Pennsylvanian reef type lacking any analogs in age (Gzhelian), size (80–100 m thick), and biodiversity (*Fomitchevella*, phylloid algae, *Ivanovia*, *Antheria*, microbialites, brachiopods, fusulines, crinoids, algae, bryozoans, *Tubiphytes*).

### Platform model

**Fig. 17:**
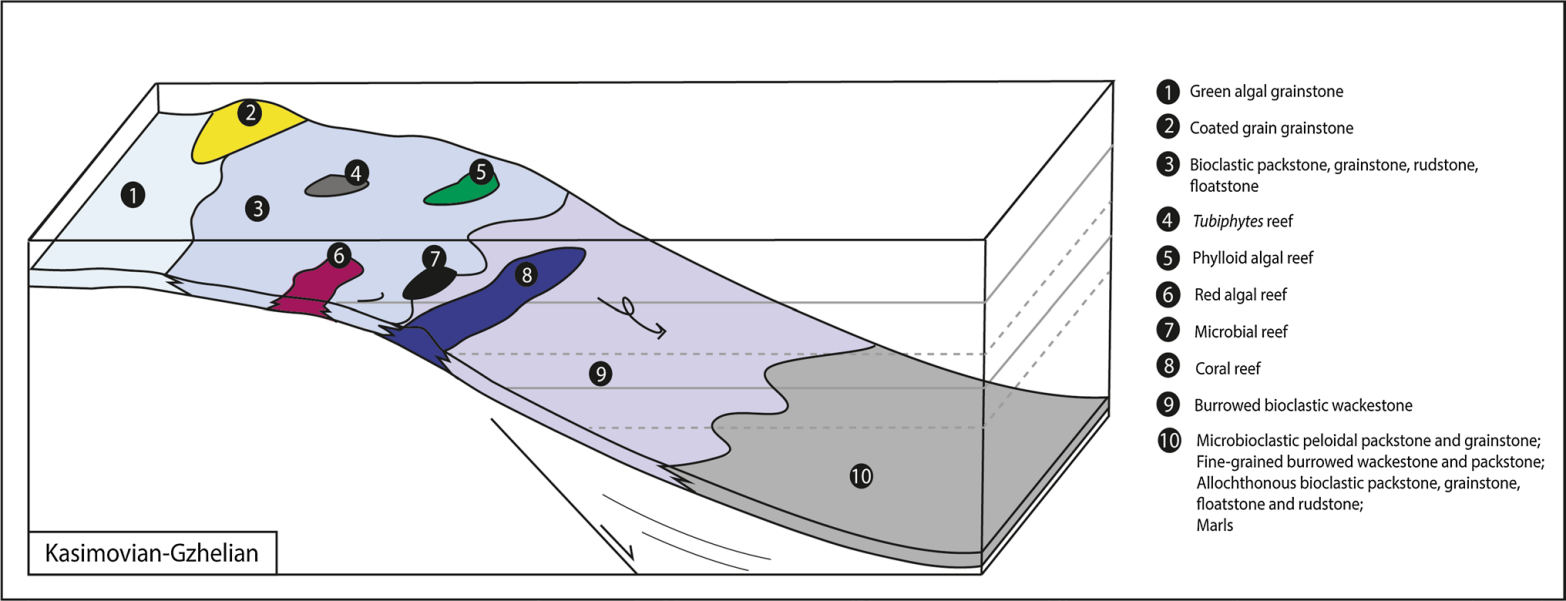
Kasimovian–Gzhelian platform model, records from Houchang (southern China). Coated-grain grainstone and green algal grainstone lithofacies types deposited in shallow waters and shoals. In situ bioclastic pack-, grain-, rud- and float-stone lithofacies type deposited around the FWWB, within the photic zone, whereas allochthonous bioclastic pack-, grain-, rud- and float-stone were reworked and deposited from peri-reefal to basinal settings. The burrowed bioclastic wackestone, microbioclastic peloidal packstone and grainstone, fine-grained burrowed wackestone and packstone and marls deposited in low energy environment, below the SWB, into the slope and basinal environment. In Houchang, phylloid algal reefs, microbial mounds, sponge mounds and *Tubiphytes* reefs grew in shallow-waters (Yao and Wang 2016). Conversely, the large Bianping coral reef was initiated in shallow-waters, mainly built by *Ivanovia,* calcimicrobes and phylloid algae, but grew with the increase of water-depth allowing the development of *Fomitchevella* branching corals, below the FWWB

The Houchang area is located at the border of a shelfal depression, inherited by pre-Carboniferous tectonic deformations (Tsien et al. 1988; Shen 2002). The Kasimovian–Gzhelian shallow-water limestones are composed of coated-grain grainstone and green algal grainstone lithofacies, deposited in shoals and/or above the FWWB. Bioclastic packstone, grainstone and rudstone deposited around the FWWB. The lack of siliciclastic deposits points to a low sediment influx, possibly due to a far distance from the Yangtze Craton. In shallow-waters, during the Kasimovian–Gzhelian, the persistence of stenohaline organisms such as bryozoans, brachiopods and foraminifera, tends to reflect normal salinity conditions (Oertli 1964; Flügel 2010). However, during the Kasimovian, the abundance of staffellids (Fig. 13), commonly considered to have wider environmental tolerance (Vachard et al. 2010), associated to sporadic schwagerinids documents possibly a time interval with slightly higher salinity, related to a restricted environment and/or semi-restricted conditions, likely controlled by sea-level fluctuations. Conversely, the Late Kasimovian–Gzhelian deep-water rocks are composed of microbioclastic peloidal packstone and grainstone, in situ fine-grained burrowed wackestone and packstone deposited in a low energy depositional environment, marls and thin allochthonous bioclastic packstone, grainstone, floatstone and rudstone layers (calciturbidites). The scarce and thin calciturbidite layers (< 1 m thick), indicate a starved basin, with a low sediment export from platform to adjacent basins. The transition from shallow-water (section 1) to basinal environments (section 2) points to a ramp, possibly distally steepened, or a slope (Fig. 17). From the Gzhelian to Early Permian (Asselian–Sakmarian), the deepwater rock succession presents a shallowing-upward sequence, dominated by the deposition of bioclastic packstone, grainstone and rudstone layers, which seems to indicate that the shelfal depression gradually became infilled, certainly due to the global sea-level fall (Early Permian glaciation; Fielding et al. 2008a; Rygel et al. 2008).

Within the Timan-Pechora Basin (Northern Russia), elongate seafloor structures are reported during the Pennsylvanian–Early Permian, consisting of narrow structural highs separated by broad depressions (Wahlman and Konovalova 2002). On the top and flanks of structural highs, light-colored and commonly fusuline-rich bioclastic grainstone–packstone shoals and *Palaeoaplysina* bioherms were deposited. Conversely, in the deeper-water shelfal depression, dark-colored argillaceous fenestrate bryozoan–crinoidal wackestone and packstone were accumulated. In the distal part of the shelf, near the western margin of the Ural Through, a carbonate bank grew on a slope or deep-ramp paleogeographic highs. The surrounding deep-water seafloor facies consist of dark-grey cephalopod-bearing marls, shales and siltstones (Wahlman and Konovalova 2002).

The sedimentary depositional systems of the Dian–Qian–Gui Basin (southern China) and Timan-Pechora Basin (Northern Russia) appear to be similar in terms of lithofacies and structures. However, the reported respective reefs differ in composition. In southern China, shallow-water reefs are composed of calcimicrobes (Huang et al. 2019), red algae (Tan 1991; Yao and Wang 2016), phylloid algae (Gong et al. 2007a, b), *Tubiphytes* (Chang et al. 2008; Guan et al. 2010; Yao and Wang 2016) and corals (Zhang et al. 2010; Gong et al. 2012), whereas in the Timan-Pechora Basin bioherms are composed of *Palaeoaplysina* and bryozoan-*Tubiphytes* (Wahlman and Konovalova 2002). The bioherm compositions can be explained by the paleolatitudes: southern China was located in subequatorial position (15°S), whereas Northern Russia was located in subtropical to temperate paleolatitudes (20–30°N; Webb 2002). Therefore, the Dian–Qian–Gui Basin (southern China) can be considered as a warm-water counterpart of the temperate Timan-Pechora Basin (Northern Russia).

## Discussion

The scarcity of Pennsylvanian coral reefs documents unfavorable conditions globally for their development. However, the exceptional occurrence of the Bianping coral reef, reported in southern China, reveals specific settings favoring the development of coral communities. This occurrence raises several questions: How do environmental conditions influence the reef communities? Which global parameters could explain the limited coral reef development? And which specific setting could have led to the development of the Bianping coral reef, despite the generally unfavorable conditions globally?

### Paleoclimate: which impact on coral reef communities?

#### Bashkirian–Moscovian

The Early Pennsylvanian (Bashkirian–Early Moscovian) coincides with a major glacial phase, with the expansion of ice-sheets in South America, southern Africa and Australia (Fielding et al. 2008a). Crowley et al. (1996) and Bruckschen et al. (1999) have estimated a tropical sea-surface temperature (SST) of about 20 ± 5 °C, 5 °C lower than that prevailing during the Viséan.

The tropical sea-surface temperature is one of the primary controlling factors in the distribution of modern tropical coral reef ecosystems (Kleypas et al. 1999; Lough 2012). Currently, reef-building corals grow optimally between 23° and 29 °C (Spalding and Brown 2015), and spend 70% of the time within a 3 °C SST range (27–30 °C; Lough 2012). Consequently, during the last decades, coral reefs occurred in the warmer part of the tropical oceans, living within 30° latitude of the equator. Most warm-water corals exhibit a symbiotic relationship with zooxanthellae algae, from which they derive much of their nutrition. However, this relationship breaks down under seawater warming, inducing episode of coral reef bleaching and mortality today (e.g. Baker et al. 2008; Hoegh-Guldberg 2011; Spalding and Brown 2015). Therefore, modern coral reefs, sensitive to seawater temperature, represent good indicators of climate changes.

Currently, even if a symbiosis between Paleozoic corals and algae is debated (Scrutton 1998; Copper and Scotese 2003), the Paleozoic coral reefs appear as adapted to soft substrates, warm waters and shelf seas (Scrutton 1998). The unequivocal evidence for this hypothesis is the radiation of the Paleozoic coral-stromatoporid reefs during the Devonian (Emsian–Givetian), the warmest period of the Phanerozoic (Copper and Scotese 2003). During this time-interval, the warm sea-surface temperature favored the formation of coral-stromatoporid mega-reefs and stretched them to high latitudes of at least 40°–50°. Based on these findings, Paleozoic and modern coral reefs have in common an affinity for warm waters. Consequently, such as modern corals, it can be assumed that Paleozoic coral reefs were also affected by climate changes, especially cooling.

**Fig. 18:**
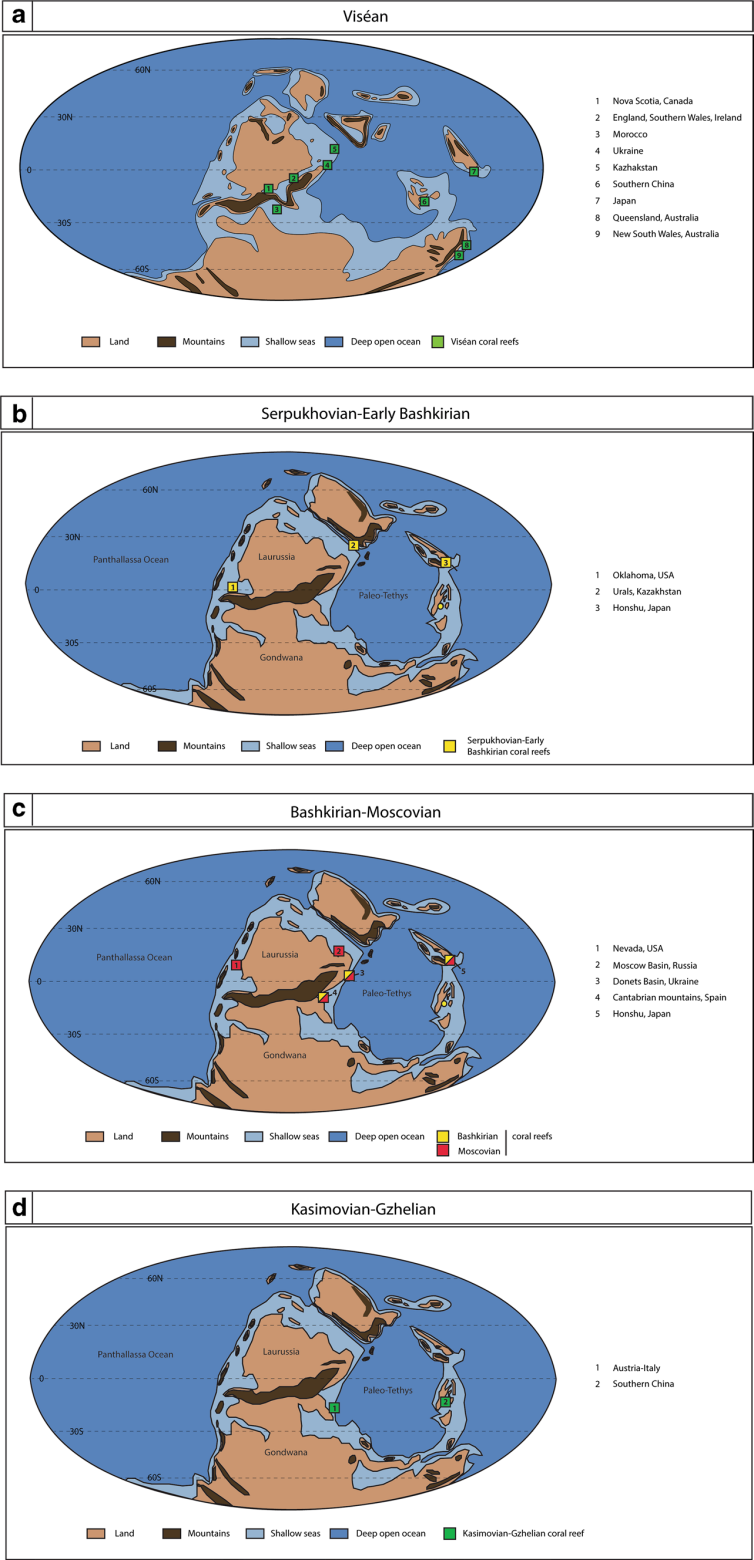
Geographical distribution of Pennsylvanian coralliferous bioconstructions plotted in Late Viséan (**a**) and Moscovian (**b**–**d**) paleogeographic maps (modified from Webb 2002). **a** Geographical distribution of Viséan coralliferous buildups. 1—Nova Scotia (Canada): Schenk et al. (1984); 2—England, southern Wales, Ireland: Wolfenden (1958), Aretz and Herbig (2003), Shen and Webb (2005), Aretz et al. (2010); 3—Morocco: Rodríguez et al. (2012); 4—Ukraine: Ogar (2012); 5—Kazakhstan: Cook et al. (1994); 6—Southern China: Gong et al. (2012), Maillet et al. (2020); 7—Japan: Ota (1968), Nagai (1985), Sugiyama and Nagai (1990,1994), Nakazawa (1997); 8—Queensland (Australia): Webb (1987, 1998), Shen and Webb (2005); 9—New South Wales (Australia): Pickett (1967), Pickett and Wu (1990). **b** Geographical distribution of Serpukhovian–Early Bashkirian coralliferous biosonstructions. 1—Oklahoma (US): Sutherland and Henry (1977); 2—Urals (Kazakhstan): Cook et al. (1994); 3—Honshu (Japan): Ota (1968), Nagai (1985), Sugiyama and Nagai (1990,1994), Nakazawa (1997). **c** Geographical distribution of Bashkirian–Moscovian coralliferous biosonstructions. 1—Nevada (US): Wilson (1963); Nelson and Langenheim (1980); Gong et al. (2012); 2—Moscow Basin: Ogar (2012); 3—Donets Basin (Ukraine): Ogar (2012); 4—Cantabrian mountains (Spain): Dingle et al. (1993), Wahlman (2002), Bahamonde et al. (2015, 2017); 5—Honshu (Japan): Ota (1968), Nagai (1985), Sugiyama and Nagai (1990,1994); Nakazawa (1997). **d** Geographical distribution of Kasimovian–Gzhelian coralliferous biosonstructions. 1—Austria–Italy: Samankassou (2003); 2—Southern China: Zhang et al. (2010); Gong et al. (2012)

The investigation of the Carboniferous coral reef distribution tends to confirm this hypothesis. Indeed, during the Viséan (Mississippian) prior to the glaciation, numerous coralliferous reefs have been reported (e.g. Australia, Pickett 1967; Pickett and Wu 1990; Webb 1987, 1998; Shen and Webb 2005; UK and Ireland, Wolfenden 1958; Adams 1984; Aretz and Herbig 2003; Aretz et al. 2010; Canada, Schenk et al. 1984; Ukraine, Ogar 2012; China, Gong et al. 2012; Yao and Wang 2016; Maillet et al. 2020; Morocco, Rodriguez et al. 2012; Kazakhstan, Cook et al. 1994), distributed from Kazakhstan to Australia, in equatorial to the subequatorial position (Fig. 18a). During the Serpukhovian and the initiation of the mid-Carboniferous glaciation, a clear decline of coralliferous reefs is observed (Fig. 18b) and the rare surviving coral reefs (e.g. Kazakhstan and USA) disappear during the Early Bashkirian, except in Japan. The mid-Carboniferous coral reef decline fairly correlated with the mid-Carboniferous climate transition, from the greenhouse to icehouse conditions. This correlation suggests that Paleozoic corals were most likely affected by cooling, and this environmental parameter played certainly a key role in the scarcity of Early Pennsylvanian coral reefs.

During the Bashkirian–Moscovian, new coral reefs developed in the Donets Basin (Ukraine), Cantabrian Mountains (Spain) and Moscow Basin (Russia) (Fig. 18c). All of these Early Pennsylvanian coralliferous reefs grew in lower latitudes, in the warmer part of the ocean, at the borders of the Paleo-Tethys Ocean and at the eastern border of the Panthalassa Ocean. The range of latitudes is significantly narrower than that of the Viséan, and climate cooling appears the most likely cause.

#### Kasimovian–Gzhelian

During the Late Pennsylvanian (Late Moscovian–Early Gzhelian), a relative climate warming occurred (Fielding et al. 2008a). While glacial retreat is observed in some regions (e.g. eastern Australia), other areas record continuing or intermittent glacial conditions (e.g. Oman, India, western Australia; Fielding et al. 2008a). However, despite the favorable climate warming, this period records a subordinate decline of coralliferous reefs, with only a few examples reported (e.g. Austria–Italy, southern China, Fig. 18d; Table 3). Therefore, paleoclimate can hardly explain the distribution of the Late Pennsylvanian coralliferous reefs, which leads to explore additional inhibiting factors for this period.

### Late Pennsylvanian: could biological competition explain the scarcity of coral reefs?

The Late Pennsylvanian (Kasimovian–Gzhelian) is characterized by sea-level fluctuations of high amplitude, varying from 10 to ~ 120 m (Ross and Ross 1987, 1988; Maynard and Leeder 1991; Haq and Schutter 2008; Rygel et al. 2008; Davydov et al. 2012), with a periodicity within the Milankovitch ranges (Harrison et al. 1979; Ross and Ross 1987; Heckel et al. 2007). These environmental conditions promoted the proliferation of phylloid algae, becoming the most abundant shallow-waters reef builders until the Permian. This radiation is attributed to the rapid reproduction and growth rates of phylloid algae, which enabled them to colonize shallow-water areas rapidly and outcompete other organisms for optimum living space. This opportunistic growth strategy was ideally suited to the cyclic glacioeustasy of the Pennsylvanian–Early Permian, which caused shallow-shelf areas to be repeatedly exposed and drowned at relatively frequent intervals (Wahlman 2002). Therefore, shallow-water shelf and shelf-margin ecological niches were newly occupied by phylloid algae (Wilson 1975), to the detriment of less adaptative and less opportunistic corals, making their recovery difficult to succeed.

In southern China, corals and phylloid algae do not occur in the same horizons (Fig. 13). Within the Bianping coral reef, phylloid algae contributed to the initiation of the reef-building under shallow and agitated waters (above or around the FWWB). However, with the increase of water depth, phylloid algae disappeared and gave way to brachiopods and branching colonial corals. Thus, the present study demonstrates that coral communities were able to colonize a deeper water setting, where they were not in competition with the contemporaneous opportunistic phylloid algal community that dominated shallower water environments. This coral growth strategy could explain the discovery of the Bianping coral reef in southern China and possibly the auloporid coral mounds reported in the Carnic Alps (Austria–Italy), growing in low-energy environments. However, the scarcity of coral reefs worldwide, even in deeper environments, leads to explore additional inhibiting factors.

### Seawater chemistry

#### Pennsylvanian aragonite seas

**Fig. 19:**
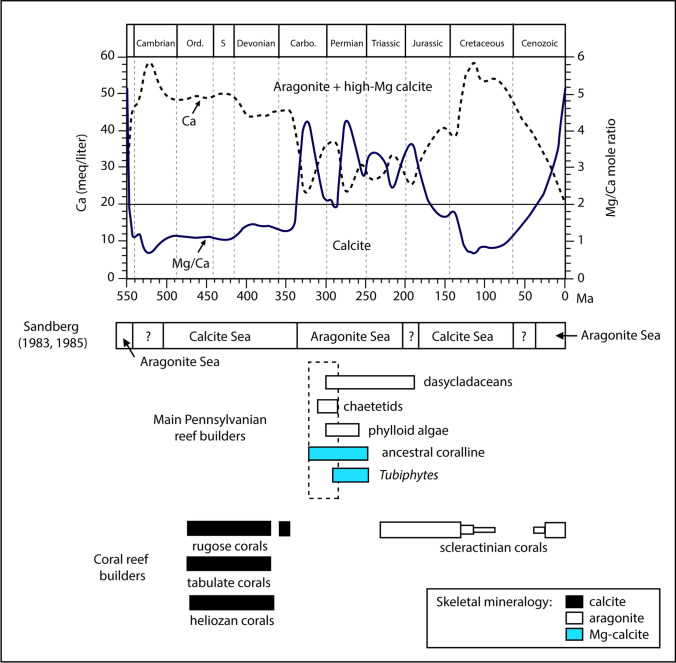
Phanerozoic secular oscillations in seawater chemistry (Stanley and Hardie 1998), between aragonite and calcite seas, favoring the precipitation of either high-Mg calcite and aragonite or low-Mg calcite. The boundary between the nucleation fields of low-Mg calcite and aragonite/high-Mg calcite is at Mg/Ca = 2. During the Pennsylvanian aragonite seas, reef builders are dominated by chaetetids, phylloid algae, ancestral coralline and *Tubiphytes*, precipitating aragonite and high-Mg calcite skeletons. System boundaries have been updated using McArthur et al. (2012). *Carbo* carboniferous, *Ord* ordovician, *S* silurian

Throughout the Phanerozoic, seawater had oscillated between aragonite and calcite seas, favoring the precipitation of either high-Mg calcite and aragonite or low-Mg calcite (Sandberg 1983; Fig. 19). The intervals of calcite and aragonite production are thought to be caused primarily by secular variations in the Mg/Ca ration of seawater (e.g. Stanley and Hardie 1998). These oscillations tend to correspond to changes in the mineralogy of abiotic as well as biotic carbonates (Stanley and Hardie 1998, 1999; Ries et al. 2006; Kiessling et al. 2008). For example, in the Early and Mid-Paleozoic calcite seas (Ordovician–Mississippian), reefs were dominated by calcitic tabulate, heliolitid, rugose corals and calcitic stromatoporoid sponges (Wood 1987). Conversely, in the Late Paleozoic–Early Mesozoic aragonite seas, reef-builders were dominated by phylloid algae (Pennsylvanian–Permian), scleractinian corals (Early Mesozoic) and high-Mg calcite ancestral coralline algae (Stanley and Hardie 1998). Therefore, the fluctuations of seawater composition impacted biocommunities, constraining them to evolve (e.g. Hautmann 2006) or disappear.

In the aragonite seas of the Pennsylvanian, shallow-water buildups are dominated by aragonitic components (e.g. phylloid algae, chaetetid sponges, aragonite cement) and high-Mg calcite skeletons (e.g. *Archaeolithophyllum* red algae, *Tubiphytes*) and cements (e.g. radiaxial; Wendt 1977; Wray 1977; Stanley and Hardie 1998). Low-Mg calcite components (e.g. bryozoan), also present, are subordinate (Wahlman 2002).

In Houchang (southern China), Late Pennsylvanian (Kasimovian–Gzhelian) bioconstructions were built by chaetetid sponges, phylloid algae, *Tubiphytes* and corals (Table 1). Globally, the poor microstructural preservation of chaetetid sponges and phylloid algae points to an original aragonitic composition (Stanley and Hardie 1998; Flügel 2010). *Tubiphytes* secreted high-Mg calcite (Senowbari-Daryan and Flügel 1991). Conversely, the mineralogy of Paleozoic corals is more debated. Sorauf (1996) argues in favor of low-Mg calcite skeletons whereas Scrutton (1997) suggests that rugose and tabulate corals secreted intermediate-Mg calcite (< 8 mol% MgCO3), and Heterocorallia precipitated low-Mg calcite skeletons. Other authors postulated an evolution of coral mineralogy through the Paleozoic: Devonian rugose corals had low-Mg calcite skeletons (*Tabulophyllum*: 0.5 weight% MgO, Sorauf 1997) whereas Pennsylvanian corals (*Lophophyllum*) precipitated original intermediate-Mg calcite skeletons (5–8 mol% MgCO_3_, Brand 1981; Webb and Sorauf 2002). The evolution of the Paleozoic coral mineralogy is attributed to the seawater chemistry and the transition from calcite to aragonite seas. Based on this assumption, all coral genera were certainly not able to evolve and most disappeared during the transition in seawater chemistry which could partly explain the decline of the coralliferous bioconstructions during the mid-Carboniferous.

This theory would suggest that all Pennsylvanian coral reef genera, including the rugose corals *Yuanophylloides*, *Axolithophyllum, Monophyllum, Caninia, Amandophyllum, Caniniostrotion, Tschussovskenia, Petalaxis, Fomitchevella* and *Cladochonus*, *Syringopora,* and tabulate coral *Multithecopora* (Table 3), precipitated intermediate-Mg calcite skeletons. However, this hypothesis is currently not demonstrated and it cannot be excluded that some of these corals precipitated low-Mg calcite skeletons.

### How could the coexistence of aragonite and high-Mg calcite components with low-Mg calcite corals be explained?

From the Early to Late Pennsylvanian, a brief drop in Mg/Ca ratio of seawater is recorded, from about 4.2–2 mol (Stanley and Hardie 1998; Fig. 19). In theory, this decline could promote the coexistence of aragonite, high-Mg calcite and low-Mg calcite skeletons during the Kasimovian–Gzhelian. However, several bioconstructions, including calcite corals, have been also reported from the Early Pennsylvanian (Bashkirian–Moscovian, Table 3), when the Mg/Ca ratio of seawater was high (about 4.2 mol), which seems to invalidate this first hypothesis.

Other studies (e.g. Adabi 2004) reveal that, even if biotic and abiotic carbonates follow the general trend of calcite seas and aragonite seas (Sandberg 1983), a coexistence of organisms with calcite and aragonite mineralogy is possible, depending on environmental conditions. In the “aragonite seas”, the precipitation of high-Mg calcite and aragonite would be favored in low latitudes and shallow-waters. Conversely, the precipitation of low-Mg calcite biota and cements would be possible thanks to cooling, decreasing of light, high paleolatitudes or increasing paleobathymetry (Wahlman 2002; Adabi 2004). This assumption is currently used to explain the occurrence of the large Viséan–Moscovian reef complex in the tropical seas of Japan. The abundance of corals in the Akiyoshi reef complex is currently attributed to the location: it formed atoll-like reef on a seamount, where they would have been exposed to the upwelling of cold open-ocean seawater (Wahlman 2002).

However, worldwide, Early Pennsylvanian coralliferous bioconstructions were mainly present in low latitudes (Fig. 18c) and shallow-waters (Table 3). Therefore, the precipitation of low-Mg calcite corals cannot be attributed to the decreasing of light, high paleolatitudes or increasing paleobathymetry. The remaining hypothesis would be climate cooling. However, this statement appears incompatible with the development of tropical corals, which raises new questions about the factors controlling this coexistence.

### How can the existence of the Late Pennsylvanian Bianping coral reefs be explained?

During the Late Pennsylvanian, the main factor inhibiting the development of coral buildups was probably biological competition with calcareous algal communities, and seawater chemistry might also have played a role. During this period, only two coral reefs have been reported in Austria–Italy and southern China. It is interesting to note that both reefs occur in a low-energy depositional environment, below the FWWB. This depositional environment differs from that of the Early Pennsylvanian, where coral reefs grew commonly in shallow waters, above the FWWB. Therefore, the deeper depositional environment where the competition of corals with phylloid algae is reduced most likely favored the development of coral communities building the large reef of the present study.

## Conclusions

1. In Houchang (southern China), the Kasimovian–Gzhelian carbonate platform records a high diversity of bioconstructions, including *Tubiphytes reefs*, sponge reefs, phylloid algal reefs, microbial mounds and a large coral reef, growing at the margin of a shelfal depression.
2. The existence of the large Bianping coral reef is uncommon for the Pennsylvanian, lacking any analogs in age (Gzhelian), size (80–100 m thick) and composition (*Fomitchevella*, phylloid algae, the corals *Ivanovia* and *Antheria*, microbialites, brachiopods, fusulines, crinoids, algae, bryozoans, *Tubiphytes*).
3. The large coral reef (southern China) was initiated in shallow waters, with the deposition of phylloid algal, the coral *Ivanovia* and microbial patch reefs. But the reef core composed of branching corals formed when water-depth increased, with the reef growth occurring below the FWWB.
4. The carbonate platform records the deposition of green algal grainstone, coated grain grainstone and bioclastic packstone, grainstone, floatstone and rudstone in shallow-waters, and burrowed bioclastic wackestone, microbioclastic peloidal packstone and grainstone, and fine-grained burrowed wackestone and packstone in the deep-water shelfal depression. In this context, the coral reef developed in the deep shelf margin, in a moderate to low energy depositional environment.
5. The investigation of the Pennsylvanian environmental conditions reveals global inhibiting factors leading to the scarcity of Pennsylvanian coral reefs:
6. The Early Pennsylvanian cooling fairly correlates with a decline of coralliferous bioconstructions. However, during the Late Pennsylvanian, despite the short-lived climate warming, this period records a subordinate decline of coral buildups. Therefore, paleoclimate could explain the collapse of the Early Pennsylvanian coral reefs but cannot explain the distribution of the Late Pennsylvanian reefs.
7. The Late Pennsylvanian coincides with the radiation of phylloid algae, more opportunistic and adaptative than corals, that occupied most of the shallow and warm waters. Consequently, the biological competition could explain the scarcity of coral reefs during the Late Pennsylvanian.
8. The Carboniferous is characterized by the transition from calcite (Mississippian) to aragonite seas (Pennsylvanian). Paleozoic corals, precipitating low to intermediate Mg calcite skeletons, were likely affected by the seawater chemistry evolution, which could explain the coral reef collapse. However, additional investigations on coral skeletons are needed to confirm this hypothesis.
9. During the Late Pennsylvanian, the main factor inhibiting the development of coral buildups was probably biological competition with calcareous algal communities, and seawater chemistry might also have played a role. However, the deeper depositional environment where the competition of corals with phylloid algae is reduced most likely favored the development of coral communities building the large reef of the present study.
